# Removal of Hexavalent Chromium in Aqueous Solution by Cellulose Filter Paper Loaded with Nano-Zero-Valent Iron: Performance Investigation and Numerical Modeling

**DOI:** 10.3390/ijerph20031867

**Published:** 2023-01-19

**Authors:** Huali Li, Zhongyu Ren, Dan Huang, Qi Jing, Haokai Tang

**Affiliations:** 1 Institute of Water Resources and Engineering, Beijing University of Technology, Beijing 100124, China; 2 Songliao Water Conservancy Commission, Songliao Basin Water and Soil Conservation Monitoring Center Station, Changchun 130021, China

**Keywords:** filter paper, nano-zero-valent iron, Cr(VI) removal, transport simulation, HYDRUS-1D

## Abstract

Cr(VI) pollution in water bodies is very harmful to human health and the environment. Therefore, it is necessary to remove Cr(VI) from water. In this study, the composite (FP-nZVI) was prepared by loading nano-zero-valent iron (nZVI) onto cellulose filter paper (FP) using a liquid-phase reduction method to improve the dispersibility and oxidation resistance of nZVI. In batch experiments, the effects of iron loading of FP-nZVI, initial concentration of Cr(VI), temperature, and pH on Cr(VI) removal were particularly investigated. The maximum removal rate of 98.6% was achieved at 25 °C, pH = 5, initial concentration of Cr(VI) of 20 mg/L, and FeCl_3_·6H_2_O solution concentration of 0.8 mol/L. The removal of Cr(VI) by FP-nZVI conformed to a pseudo-second-order kinetic model and Langmuir isotherm model. The mechanism of Cr(VI) removal was a multi-step removal mechanism, involving adsorption, reduction, and coprecipitation. Column experiments investigated the effect of flow rate (1 mL/min, 3 mL/min, and 5 mL/min) on Cr(VI) removal. We found that increasing flow rate slightly decreased the removal rate of Cr(VI). The transport of Cr(VI) in composite porous media was simulated using HYDRUS-1D, and the results show that the two-site model can well simulate the reactive transport of Cr(VI). This study may provide a useful reference for the remediation of groundwater contaminated with Cr(VI) or other similar heavy metals using FP-nZVI.

## 1. Introduction

Chromium contamination in water is extremely dangerous to human health and ecosystems. Cr(VI) has greater solubility, mobility, and toxicity than Cr(III). The treatment methods for Cr(VI) in water mainly include the biological method, ion-exchange method, membrane separation technology, adsorption method, and adsorption–reduction method. The biological method [1] mainly includes the enrichment of Cr(VI) by plants and the removal of microorganisms. Guo [2] et al. used Pseudomonas to explore its adsorption capacity for Cr(VI) and found that under the optimal conditions, the removal rate of Cr(VI) by Pseudomonas could reach 72.05%. The ion-exchange method [3] is that in which Cr(VI), which mainly exists in water in the form of oxygen anion, is separated from water by replacing it with functional groups available for reaction in anion-exchange resin. Rivero [4] et al. found that it was feasible to use ion-exchange resin Lewatit MP-64 to treat Cr(VI)-polluted groundwater. Membrane separation technology [5] relies on some kind of mass transfer power (such as pressure difference, concentration difference, or potential difference) to move each component of water selectively through the membrane, so as to achieve Cr(VI) interception. Two new positively charged ultrafiltration membranes prepared by Yao [6] et al. showed high Cr(VI) adsorption capacity at low pH. Adsorption method [7] refers to the accumulation of pollutants on the adsorbent surface to achieve the purpose of Cr(VI) removal. Using activated carbon, Chen et al. found that the highest Cr(VI) adsorption in aqueous solution (40.04%) was achieved at a pH of 3 [8]. The biological method and ion-exchange method have a long periodicity, and the maintenance cost of membrane separation technology is high. Therefore, as the synergistic effect of adsorption and reduction, the adsorption–reduction method has more competitive advantages [9] in converting toxic Cr(VI) into Cr(III) through common methods of chromium pollution remediation [10,11] and adsorbing pollutants and reaction products on the adsorbent. The NZVI-rGO prepared by Jing [12] et al. has a good removal effect on Cr(VI) in water, and they found that Cr(VI) is fixed on the surface of NZVI-rGO through electrostatic action and then reduced to Cr(III) by NZVI. Soliemanzadeh [13] et al. used bentonite loaded with nano-zero-valent iron to remove Cr(VI) and found that the bentonite adsorbed Cr(VI) and zero-valent iron reduced it to Cr(III). Compared with other methods, the adsorption–reduction method has the characteristics of low cost, convenient operation, and relatively high removal efficiency, which attracts extensive interest of researchers.

nZVI has a high specific surface area, which is conducive to the surface adsorption of pollutants, and its high redox activity can promote the decomposition of organic pollutants and the conversion of inorganic pollutants to other states [14]. nZVI has been widely used in the remediation of chromium-contaminated groundwater and soil. However, because of the high surface energy and magnetic properties of the nZVI particles, they are easy to agglomerate and oxidize when used alone, resulting in a decrease in their reactivity in applications. Previous studies have developed a series of modification measures to improve the shortcomings of nZVI, including bimetallic composites [15], surface modification [16], and solid loading [17], all of which can maintain the reactivity of nZVI. However, bimetals may be expensive and may also lead to secondary contamination [18]. Surface modifiers may reduce the number of active sites and inhibit electron transfer from ZVI to contaminants [19]. Solid loading allows synergistic effects to play a role while maintaining the benefits of bimetallic composites and surface modification [20]. According to the current literature, it is a common method to wrap nZVI particles in carriers, which include organic matters (chitosan [21], carboxymethyl cellulose [22], and sucrose [23]), clay materials (zeolite [24], kaolin [25], and bentonite [26]), and carbon materials (biochar [27], activated carbon [28], and graphene [29]). Most of these materials are tiny spheres or powders [30], which are inconvenient to remove or easily form sludge, and some may even cause secondary pollution. Therefore, it is necessary to select an insoluble and environment-friendly carrier with good supporting capacity and without blocking the flow system to immobilize the nZVI particles.

Cellulose-based compounds as structural support materials and adsorbents have garnered interest due to their biodegradability, sustainability and strong mechanical properties [31]. FP is cheap and easy to obtain and transport. It contains oxygen-rich lined microfibrils and pores. Its overall structure provides anchoring positions and internal cavities for trapping metal ions and metal nanoparticles [32]. Yu [33] et al. prepared polyacrylic-acid-modified filter paper, which was used as a matrix to chelate nZVI, and the results show that the decolorization rate of methyl blue and methylene blue can reach more than 95%. Kamal [34] et al. synthesized highly active copper nanoparticles in chitosan coating layers over cellulose microfibers of filter paper, which have an important catalytic effect on the reduction of methyl orange and Congo red. As a result, FP can be considered as a good carrier for nanoparticles.

In this study, as the carrier of nZVI particles, FP has a rich porous structure, which can provide an anchor position to limit the release of nZVI particles from FP to the water, avoiding secondary pollution. Commonly, the preparation of composite materials loaded with nZVI by liquid-phase reduction requires continuous input of nitrogen, but in this study, nitrogen is not required, and the supported FP-nZVI system with strong oxidation resistance, good dispersion, and good Cr(VI) removal ability can also be obtained. The preparation process of FP-nZVI is convenient for transportation, storage, and application, and it can provide new ideas for site remediation. The FP-nZVI was characterized by Fourier transform infrared spectroscopy (FTIR), scanning electron microscopy (SEM), X-ray diffraction (XRD), X-ray photoelectron spectroscopy (XPS), and Brunauer–Emmett–Teller (BET). The removal effect of FP-nZVI on Cr(VI) in water was investigated by batch and column experiments. The transport of Cr(VI) in a porous medium composed of FP-nZVI and sand was simulated by HYDRUS-1D. The aims of this study are to design a filter paper composite system loaded with nZVI nanoparticles with good dispersion and antioxidant stability through a convenient preparation process and explore its performance and mechanism of Cr(VI) removal.

## 2. Materials and Methods

### 2.1. Materials

All reagents used in this study were analytically pure and used as received without further purification. FP (Grade 40, radius 55 mm) was purchased from Whatman. Chemical reagents included ferric chloride hexahydrate (FeCl_3_·6H_2_O), anhydrous ethanol (C_2_H_6_O), sodium borohydride (NaBH_4_), diphenylcarbonyl dihydrazide (C_13_H_14_N_4_O), acetone (CH_3_COCH_3_), potassium dichromate (K_2_Cr_2_O_7_), o-phenanthroline (C_12_H_8_N_2_), and anhydrous sodium acetate (CH_3_COONa). Above chemical reagents were produced by Tianjin Fuchen Chemical Reagent Factory. Sodium hydroxide (NaOH), hydrochloric acid (HCl), sulfuric acid (H_2_SO_4_), and phosphoric acid (H_3_PO_4_) were produced by Beijing Chemical Factory.

### 2.2. Preparation of FP-nZVI

In the experiment, 1.0812, 2.1624, 5.4059, 8.6495, and 10.8119 g FeCl_3_·6H_2_O were dissolved in five parts of 40 mL ethanol solvent to prepare iron solutions with different concentrations (0.1, 0.2, 0.5, 0.8, and 1.0 mol/L). Five parts of 0.02 g NaOH were dissolved in 100 mL of deionized water, and twenty pieces of filter papers were divided into five equal portions and immersed in the solution simultaneously. After 30 min, the filter papers were removed and washed with deionized water until the deionized water was approximately neutral; the washed filter papers were immersed in the iron solution for 40 min and then taken out and air-dried to obtain yellow filter papers. Finally, five parts of 0.95 g NaBH_4_ were weighed and dissolved in five parts of 100 mL deionized water to prepare five parts of the reducing agent. The five parts of yellow filter papers were immersed directly into the NaBH_4_ solution for 45 min to produce black filter papers (FP-nZVI). The FP-nZVI composites were thoroughly cleaned with deionized water and anhydrous ethanol and finally dried under vacuum for 600 min in a freeze-drying oven (Shanghai Bilan Instrument Manufacturing Co., Ltd., FD-1A-50, Shanghai, China).

### 2.3. Characterization

The nanoscale morphology of FP-nZVI was obtained by SEM (JEOL, JEM-7001M, Tokyo, Japan) at an operating voltage of 10 kV. The surface functional groups of FP-nZVI were detected using an FTIR analyzer (Shimadzu, IRAffinity-1S, Tokyo, Japan). The crystal structures of FP-nZVI were analyzed by Bruker D8 Advance XRD instrument. The surface element composition and valence states of FP-nZVI were obtained by XPS (Thermo Fisher, ESCALAB-250Xi, Waltham, MA, USA). The BET surface areas of FP and FP-nZVI were determined by adsorption–desorption instrument (Quantachrome, Autosorb-iQ, Boynton Beach, FL, USA).

### 2.4. Batch Experiments

A total of four series of batch analyses were conducted to investigate the effects of different Fe^3+^ concentrations used to prepare FP-nZVI, initial Cr(VI) concentration, temperature, and pH on the removal of Cr(VI). The reaction conditions are shown in Table 1.

Batch experiment 1 was to investigate the effect of FP-nZVI prepared with different Fe^3+^ concentrations on the removal effect of Cr(VI). Five 250 mL triangular flasks were filled with 200 mL of Cr(VI) solution at a concentration of 20 mg/L, and the Cr(VI) solution was deoxygenated with N_2_ for 20 min. Then, one piece of FP-nZVI was put into each triangular flask filled with Cr(VI) solution. (Five pieces of FP-nZVI used had been prepared with FeCl_3_·6H_2_O solutions of 0.1 mol/L, 0.2 mol/L, 0.5 mol/L, 0.8 mol/L, and 1.0 mol/L, respectively.) With initial pH = 5, sealed with sealing film, and keeping the temperature at 25 °C, the triangular flasks were placed in a constant-temperature shaker (Shanghai Bilang Company, COS-100B, Shanghai, China) and shaken at 150 r/min. Samples were taken by syringe at 2, 5, 10, 20, 30, 60, 90, 120, 150, 180, and 210 min. The concentrations of Cr(VI) were determined by diphenylcarbonyl dihydrazide spectrophotometric method (GB7467-87) after passing through 0.22 µm filter membrane.

Batch experiment 2 was to investigate the effect of initial concentration of Cr(VI) on the removal effect of Cr(VI). In total, 200 mL of the Cr(VI) solutions at concentrations of 5 mg/L, 10 mg/L, 20 mg/L, 30 mg/L, and 40 mg/L was added in five triangular flasks, respectively, and the Cr(VI) solution was deoxygenated with N_2_ for 20 min. Then, one piece of FP-nZVI prepared with 0.8 mol/L FeCl_3_·6H_2_O solution was put into each triangular flask. The concentration of Cr(VI) was determined after shaking in a shaker and taking samples. Initial pH, temperature, sampling scheme, and testing method were the same as batch experiment 1.

Batch experiment 3 was to investigate the effect of temperature on the removal effect of Cr(VI). Five 250 mL triangular flasks were filled with 200 mL of Cr(VI) solution at a concentration of 20 mg/L, and the Cr(VI) solution was deoxygenated with N_2_ for 20 min. Then, one piece of FP-nZVI prepared with 0.8 mol/L FeCl_3_·6H_2_O solution was put into each triangular flask. The initial pH = 5 was adjusted, and the control temperatures were kept at 15 °C, 20 °C, 25 °C, 30 °C, and 35 °C. The concentration of Cr(VI) was determined after shaking in a shaker and taking samples. The sampling scheme and testing method were the same as batch experiment 1.

Batch experiment 4 was to investigate the effect of initial pH on the removal effect of Cr(VI). Five 250 mL triangular flasks were filled with 200 mL of Cr(VI) solution at a concentration of 20 mg/L, and the Cr(VI) solution was deoxygenated with N_2_ for 20 min. Then, one piece of FP-nZVI prepared with 0.8 mol/L FeCl_3_·6H_2_O solution was put into each triangular flask. The initial pH was adjusted to 3, 5, 7, 9, and 11, and the temperature was controlled at 25 °C. The concentration of Cr(VI) was determined after shaking in a shaker and taking samples. The sampling scheme and testing method were the same as batch experiment 1.

Parallel experiments were set up for all experimental procedures, and the experimental results were averaged to reduce accidental errors.

The removal rate is calculated by Equation (1):

(1) $$\mathrm{Removal}\;\mathrm{rate}\left(\%\right)=\left(C_{0}-C_{t}\right)/C_{0}\times 100\%$$

where C_0_ is the initial Cr(VI) concentration at t = 0 and C_t_ is the remaining Cr(VI) concentration in the solution at time t.

At 210 min of reaction, the removal rates of Cr(VI) by FP-nZVI prepared with 0.1, 0.2, and 0.5 mol/L iron concentration were 66.97%, 80.04%, and 98.22%, respectively. When the iron concentration was 0.8 and 1.0 mol/L, the removal rate of Cr(VI) had reached 98% at 30 min of reaction.

Since the removal effect of FP-nZVI on Cr(VI) was essentially the same for FP-nZVI prepared with iron solution concentrations of 0.8 mol/L and 1.0 mol/L, FP-nZVI prepared with iron solution concentration of 0.8 mol/L was used for subsequent experiments. These experiments included FTIR, SEM, XRD, and XPS analysis; the study of effects of initial concentration, reaction temperature, reaction pH, and aging time on the removal of Cr(VI); and the isothermal adsorption analysis, removal mechanism analysis, and column experiments.

### 2.5. Fe Content Measurement

First, 60 mL of 1:2 hydrochloric acid solution was measured in a triangular flask. Then, one piece of FP-nZVI was put into the triangular flask. The triangular flask was sealed with sealing film, and the temperature was kept at 25 °C. The flask was placed in a constant temperature shaker and shaken at 150 r/min for one hour to make the iron powder in FP-nZVI come off completely. Then, 1 mL of the solution was taken to be measured in a 100 mL colorimetric tube, and the iron loading of FP-nZVI was determined according to GB/T3049-2006 1,10-phenanthroline spectrophotometric method. Repeating the above procedure, the iron loading of FP-nZVI prepared by FeCl_3_·6H_2_O solution with concentrations of 0.1 mol/L, 0.2 mol/L, 0.5 mol/L, 0.8 mol/L, and 1.0 mol/L could be determined.

### 2.6. Column Experiments

**Figure 1:**
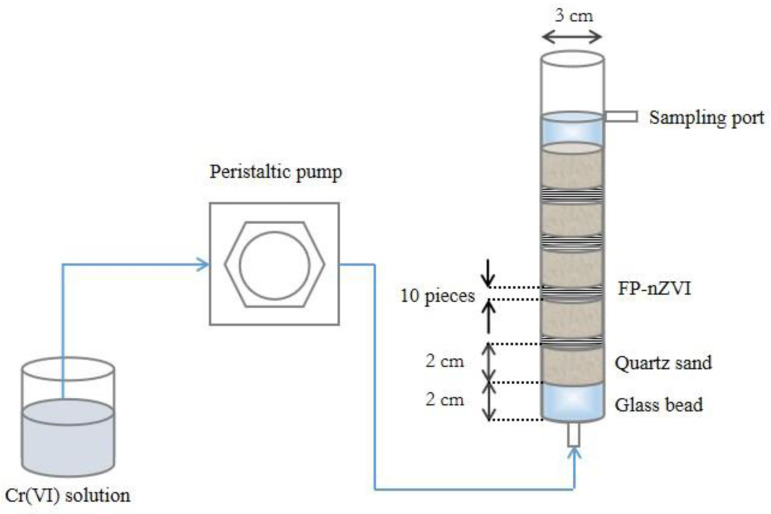
Schematic diagram of column experimental device.

The column experimental device consisted of a Plexiglas cylinder (internal diameter = 3 cm, thickness = 0.3 cm, height = 23 cm), a storage container for Cr(VI) solution, a peristaltic pump, and a hose for solution delivery. The Plexiglas cylinder was filled with acid-washed quartz sand, FP-nZVI, and glass beads, with a total filling height of 18 cm, and the filling position of each material is shown in Figure 1. The quartz sand was purchased from Shanghai Macklin Biochemical Technology Co., Ltd. (Shanghai, China), with a particle size of 0.55–1 mm. The glass beads were purchased from Beijing Mreda Technology Co., Ltd. (Beijing, China), with a particle size of 3–4 mm.

The peristaltic pump used in the experiment was BT100-1L-type (Baoding Lange Constant Flow Pump Co., Ltd., Baoding, China); the storage container for Cr(VI) solution was a 1L volumetric flask. The material of the hose for solution delivery was silica gel.

Each time before formal injection of Cr(VI) solution, deionized water was injected through the bottom of the column with a peristaltic pump to saturate the whole medium in the column and achieve a stable flow state. Then, deionized water was replaced with Cr(VI) solution. Cr(VI) solution with initial pH = 5 and initial concentration of 20 mg/L was continuously injected at flow rates of 1 mL/min, 3 mL/min, and 5 mL/min. Samples were taken at certain time intervals at the sampling port, and the concentration of Cr(VI) was determined.

In addition, the porosity and permeability coefficient of experimental medium were measured. Using the conservative substance Cl^−^ as a tracer, transport experiments were performed to obtain the dispersion coefficient of the medium. See Text S1–S3 and Table S1–S3 for details. These parameters were used for subsequent numerical simulations of Cr(VI) in the intracolumn medium.

## 3. Results and Discussion

### 3.1. Characterizations

#### 3.1.1. FTIR Analysis

**Figure 2:**
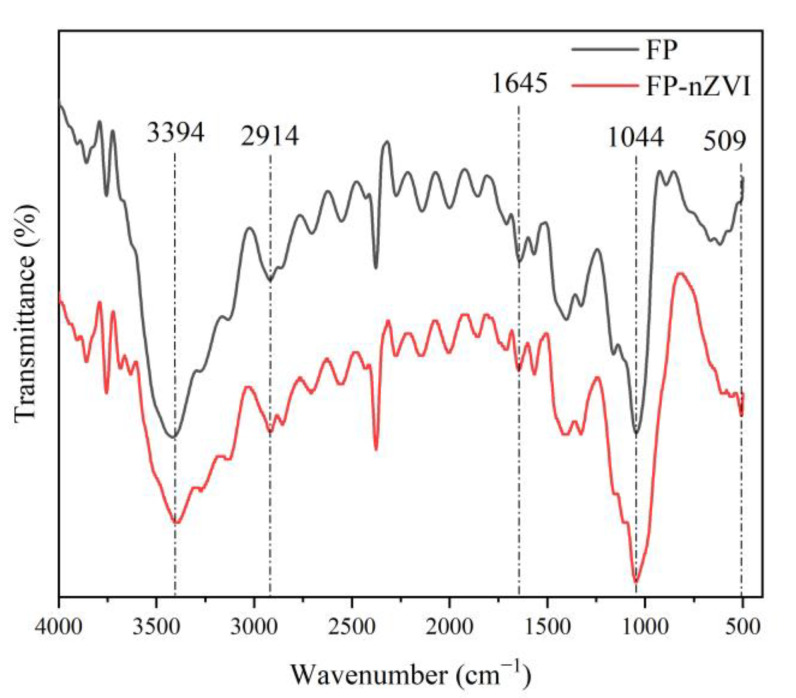
FTIR spectra of FP and FP-nZVI.

FTIR analysis was performed for FP and FP-nZVI, and the results are shown in Figure 2. In Figure 2, 3394 cm^−1^ is the stretching vibration peak of -OH; 2914 cm^−1^ is the stretching vibration peak of -CH_2_; 1645 cm^−1^ can be attributed to H-O-H stretching [35]; and 1044 cm^−1^ is the C-O stretching vibration peak [36]. When nZVI was loaded onto FP, a new peak of FP-nZVI appeared at 509 cm^−1^, which can be attributed to Fe-O stretching vibration [37]. Thus, FTIR spectra indicated that nZVI was successfully loaded on the FP.

#### 3.1.2. SEM Analysis

**Figure 3:**
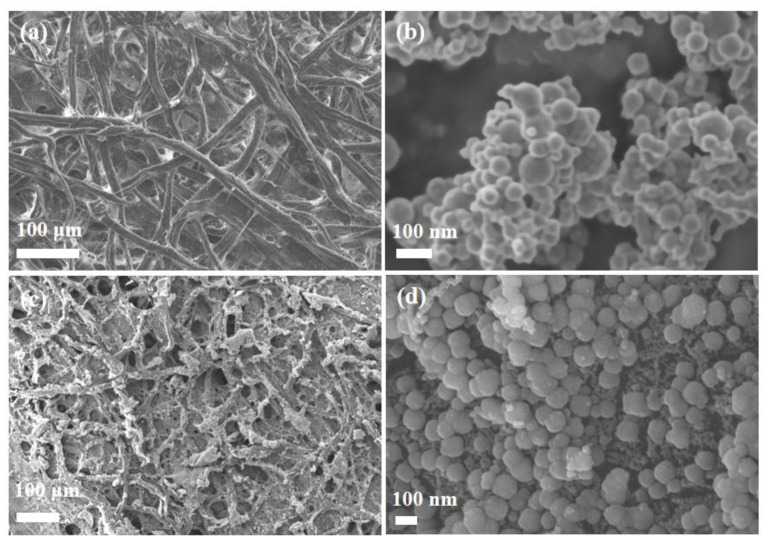
SEM images of (**a**) FP, (**b**) nZVI, (**c**) FP-nZVI; (**d**) Magnification of (**c**), morphology of nZVI was presented.

The SEM images of FP, nZVI, and FP-nZVI are shown in Figure 3. Figure 3a shows the SEM image of FP. It can be seen that FP presented a porous microstructure, and its fiber strip surface was slightly rough, which was conducive to the loading of nZVI. Moreover, the entry of nZVI into the inner space of the fiber channel can effectively avoid oxidation. Figure 3b shows a clear agglomeration of bare nZVI, which was caused by van der Waals forces and magnetic effects between the nZVI particles [38]. Figure 3c,d is the SEM images of FP-nZVI. It can be seen that the iron nanoparticles of FP-nZVI existed as separately dispersed and the particles were regular spheres with a particle size of about 100 nm. The reason was that the hydroxyl groups rich in cellulose filter paper fibers had a strong interaction with nZVI [31], which prevented the large-scale agglomeration of nZVI particles. Wang et al. also found that the presence of hydroxyl groups on the membrane surface can significantly improve iron loading [39].

#### 3.1.3. XRD Analysis

**Figure 4:**
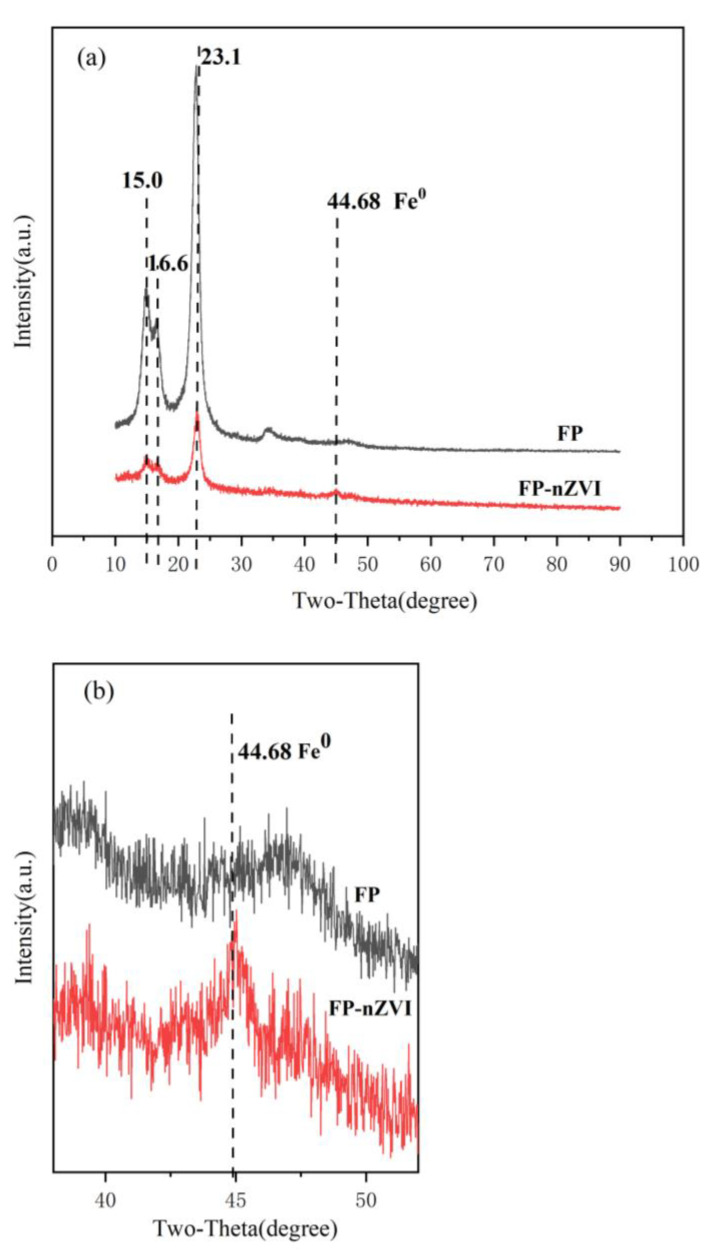
(**a**) XRD pattern of FP and FP-nZVI; (**b**) Magnification of (**a**).

Figure 4 shows the XRD diffraction patterns of FP and FP-nZVI with 2θ of 15.0°, 16.6°, and 23.1°, which stand for the (1$\bar{1}$0), (110), and (200) diffraction peaks of cellulose, respectively [40]. Diffraction patterns of FP-nZVI showed a 110 crystal plane diffraction peak of α-Fe at 2θ of 44.68°, which was a typical peak of nZVI [41], indicating that the prepared iron existed in the form of zero-valent iron. The diffraction peak of Fe^0^ was observed at 44.8° for nZVI-BC prepared by Huang et al., which was similar to the results of this study [42]. The crystallite size of FP-nZVI was calculated to be 9.06 nm by Scherrer equation, which was smaller than the SEM result (about 100 nm). The finer sizes of the primary nZVI particles can lead to the amorphous structure of the stabilized nZVI [43,44]. The Scherrer equation obtained the crystallite size, i.e., primary particle size, while SEM obtained the particle size, i.e., secondary particle size. It is generally believed that the reason why the secondary particle size was much larger than the primary particle size was due to the agglomeration and growth of crystallites [45]. Therefore, the particle size observed by SEM was larger than the crystallite size calculated by Scherrer equation. Similar results have been found in other literature [46,47].

#### 3.1.4. XPS Analysis

**Figure 5:**
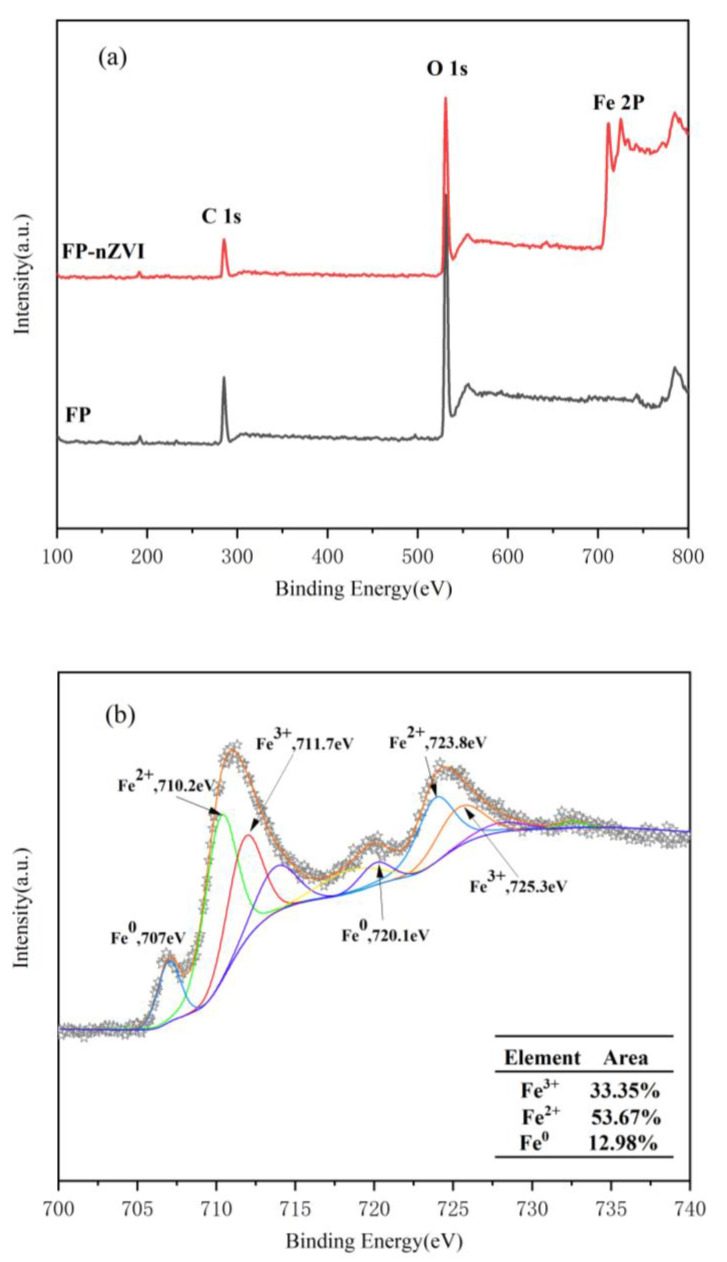
(**a**) XPS full-spectrum scan of FP and FP-nZVI; (**b**) Fe2p narrow-spectrum scan of FP-nZVI. The lines with different colors are the fitting curves of the intensity of the corresponding valence states of the iron element. The star curve is the fitting curve of the total strength curve of iron element.

Figure 5a shows the XPS full-spectrum scans of FP and FP-nZVI. Binding energies near 286.08 eV and 531.2 eV were observed in both FP and FP-nZVI, which were attributed to C1s and O1s, respectively. When the filter paper was loaded with iron, one can observe the electron binding energy Fe2p at 711.08 eV and 725.1 eV in the resolution scanning image, which was the photoelectron energy measured when the 2p orbital electrons of iron atoms were excited. In order to better explore the existing form of iron in FP-nZVI, narrow-spectrum scans of Fe2p were performed using XPS (as shown in Figure 5b). The lines with different colors are the fitting curves of the intensity of the corresponding valence states of the iron element. The star curve is the fitting curve of the total strength curve of iron element. At the binding energies of 710.2 eV and 723.8 eV, there were 2p_3/2_ characteristic peaks and 2p_1/2_ characteristic peaks corresponding to Fe^2+^, respectively. At the binding energies of 711.7 eV and 725.3 eV, there were 2p_3/2_ characteristic peaks and 2p_1/2_ characteristic peaks corresponding to Fe^3+^, respectively. These iron oxidations occurred mainly during the synthesis process and slight oxidation of the nZVI surface [48]. The characteristic peaks corresponding to Fe^0^ were found at binding energies of 707.0 eV and 720.1 eV, which proved the successful loading of nZVI particles on FP.

#### 3.1.5. BET Analysis

**Figure 6:**
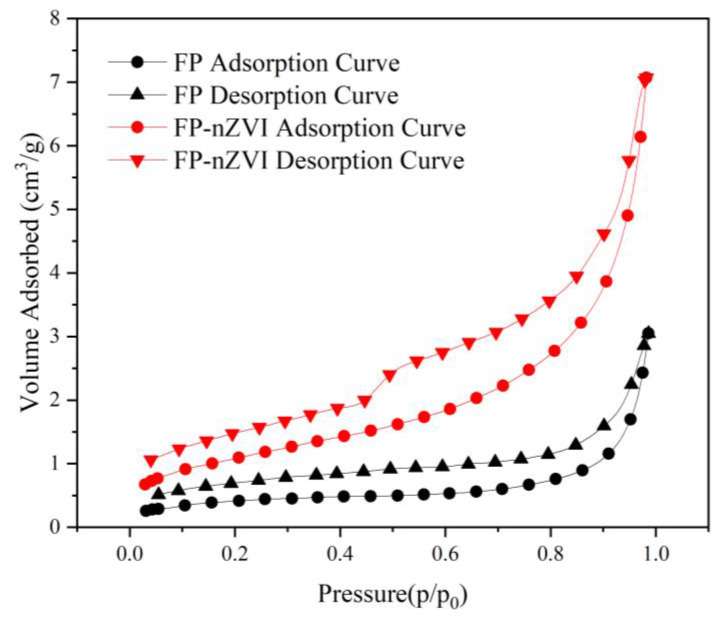
N_2_ adsorption and desorption isotherm of FP and FP-nZVI.

N_2_ adsorption–desorption curves of FP and FP-nZVI are shown in Figure 6. The specific surface areas of FP and FP-nZVI were 1.517 m^2^/g and 3.963 m^2^/g, respectively. The enlarged specific surface area of FP-nZVI can provide more active sites and contact area for the degradation of pollutants, thus improving the removal efficiency [49]. The average pore size of FP-nZVI was 5.770 nm, and the pore volume was 0.010 cc/g, indicating that the material was structurally porous and could also provide many reduction and adsorption sites for pollutants.

### 3.2. Batch Experiments

#### 3.2.1. Effect of Iron Loading on the Removal of Cr(VI)

**Figure 7:**
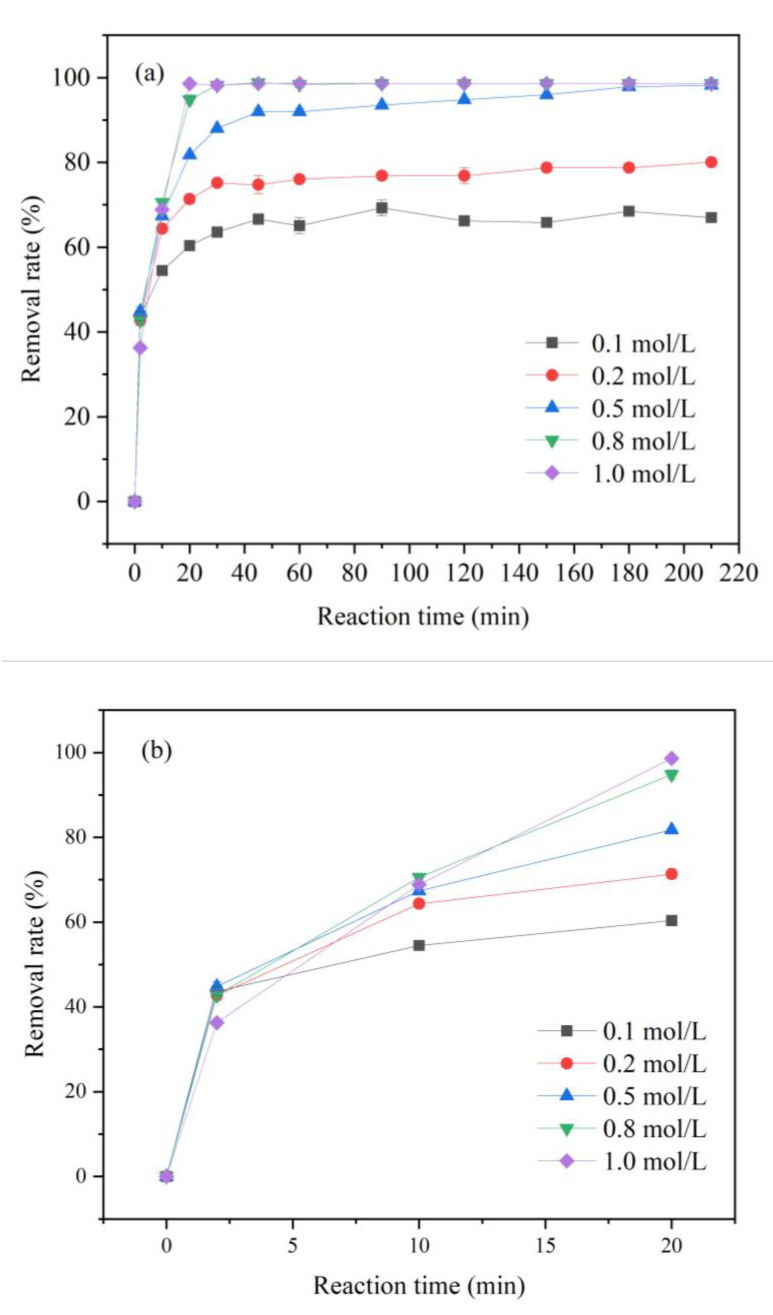
(**a**) Removal rate of Cr(VI) by FP-nZVI prepared with different iron concentrations; (**b**) Removal rate in the first 20 min.

The effects of iron loading of FP-nZVI prepared with different concentrations of ferric chloride hexahydrate solution (0.1, 0.2, 0.5, 0.8, and 1 mol/L) on the removal of Cr(VI) were investigated at 25 °C, pH = 5, and an initial concentration of Cr(VI) of 20 mg/L. The iron loadings of FP-nZVI prepared with five solutions were 55.62, 61.85, 65.28, 85.35, and 93.22 mg, respectively. As shown in Figure 7, the reaction can be divided into two stages. The first 20 min was a relatively fast reaction stage. From after 20 min to the end time, it was basically a low-speed reaction process, and it eventually reached equilibrium. The higher the initial concentration of iron solution, the higher the removal rate of Cr(VI) at equilibrium. When the concentration of iron solution was 0.1, 0.2, and 0.5 mol/L, the removal rates of Cr(VI) reached 66.97%, 80.04%, and 98.22%, respectively, at 210 min of reaction. Furthermore, when the concentration of iron solution was increased to 0.8 mol/L and 1 mol/L, the removal rate of Cr(VI) could reach 98% at 30 min of reaction. This was because as the concentration of iron solution increased, the iron loading of FP-nZVI became larger, and therefore more reaction active sites were available. At the same time, it could provide more contact reaction opportunities for Cr(VI), thus consuming more Cr(VI). As a result, the reaction rate was faster and the removal rate of Cr(VI) was improved [50].

#### 3.2.2. Effect of Initial Concentration on the Removal of Cr(VI)

**Figure 8:**
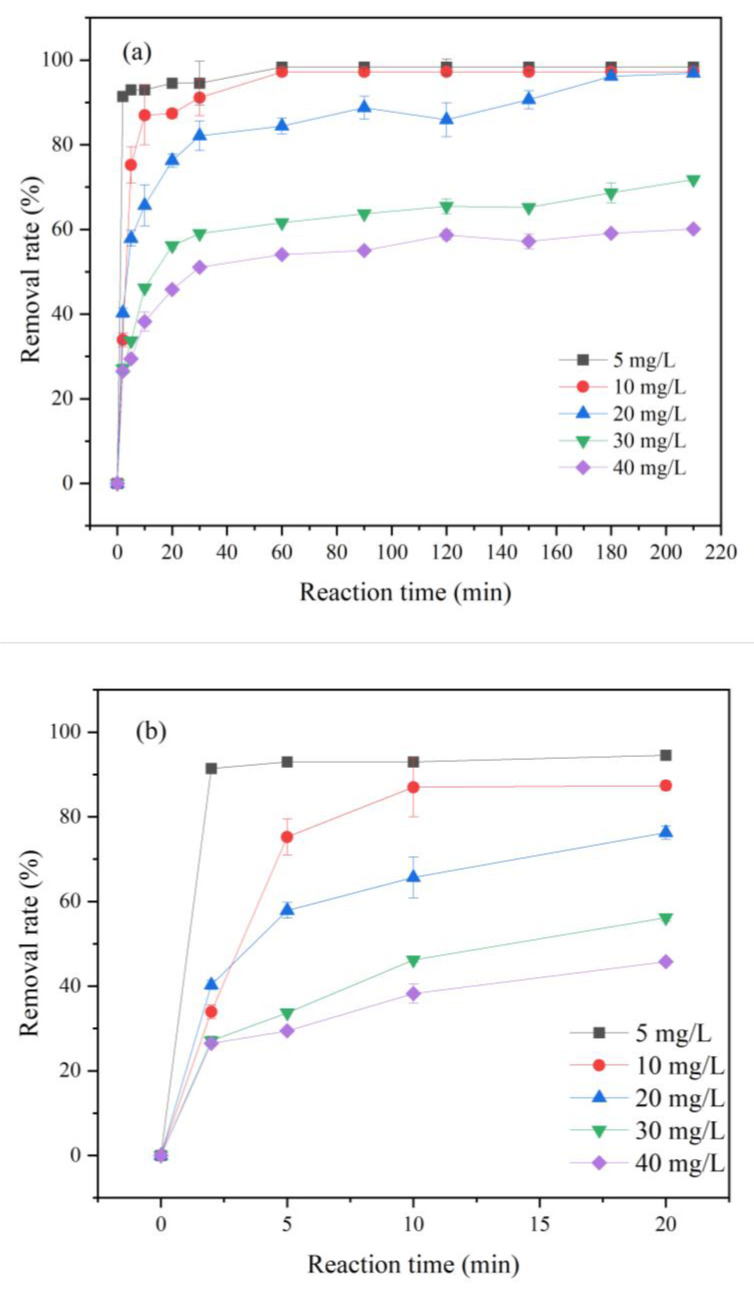
(**a**) Removal rate of Cr(VI) at different initial concentrations of Cr(VI); (**b**) Removal rate in the first 20 min.

Under the condition of 25 °C and pH = 5, the effects of different initial concentrations of Cr(VI) (5, 10, 20, 30, 40 mg/L) on the removal effect of Cr(VI) by FP-nZVI prepared by 0.8 mol/L ferric chloride hexahydrate solution were investigated. As shown in Figure 8, the larger the initial concentration of Cr(VI), the lower the removal rate. Fazlzadeh et al. also found that the removal rates of TV-Fe, UD-Fe, and RD-Fe prepared with three green plant extracts decreased with the increase in initial concentration of Cr(VI) [51]. This is consistent with the results of this study. When the initial concentration of Cr(VI) was 5 mg/L and 10 mg/L, the Cr(VI) removal rate reached more than 90% for the first 30 min of the reaction. When the initial concentration of Cr(VI) increased to 20 mg/L, the Cr(VI) removal rate was about 80% for the first 30 min of the reaction. When the initial concentration of Cr(VI) was further increased to 30 mg/L and 40 mg/L, the removal rates were only 59% and 51% for the first 30 min of the reaction. This was because the reactive active sites that FP-nZVI can provide were relatively sufficient when the Cr(VI) concentration was low. With the increase in Cr(VI) concentration, more Cr(VI) would contact and react with FP-nZVI. nZVI would be rapidly oxidized, and the active sites tended to saturate, thus reducing the reaction activity. Furthermore, the high strength of Cr(VI) would reduce the electrochemical corrosion of nZVI, limit the formation of Fe(II), and reduce the reaction rate. At the same time, the reduction of Cr(VI) was incomplete, reducing the removal rate [52].

#### 3.2.3. Effect of Reaction Temperature on the Removal of Cr(VI)

**Figure 9:**
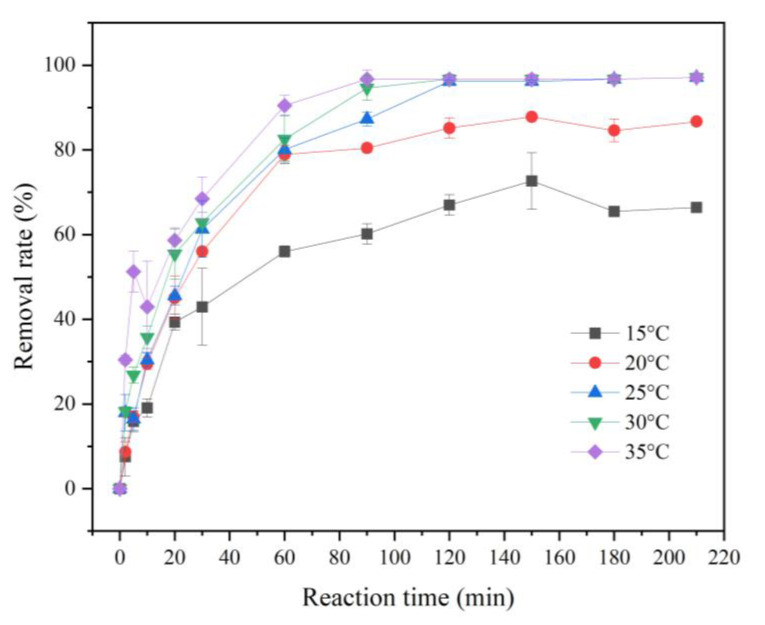
Removal rate of Cr(VI) at different reaction temperatures.

Under the condition of pH = 5 and initial concentration of Cr(VI) of 20 mg/L, the effects of different reaction temperatures (15, 20, 25, 30, 35 °C) on the removal effect of Cr(VI) by FP-nZVI prepared with 0.8 mol/L ferric chloride hexahydrate solution were investigated. From Figure 9, it can be seen that both the reaction rate and the Cr(VI) removal rate was accelerated or increased with the rising of reaction temperature. After 90 min, the removal rates of Cr(VI) at reaction temperatures of 15 °C, 20 °C, and 25 °C were 60.15%, 80.42%, and 87.23%, while the removal rates at reaction temperatures of 30 °C and 35 °C were greater than 95%. It can be seen that the increase in reaction temperature would promote the removal of Cr(VI). This was due to the reaction between nZVI and Cr(VI), which was endothermic [53]. Increasing the temperature was conducive to the positive chemical reaction, thus increasing the removal rate of Cr(VI). The increase in temperature led to a more intense movement of Cr(VI) ions in the solution, so that Cr(VI) would have a greater chance to contact and react with nZVI, thus accelerating the reaction rate.

#### 3.2.4. Effect of Reaction pH on the Removal of Cr(VI)

**Figure 10:**
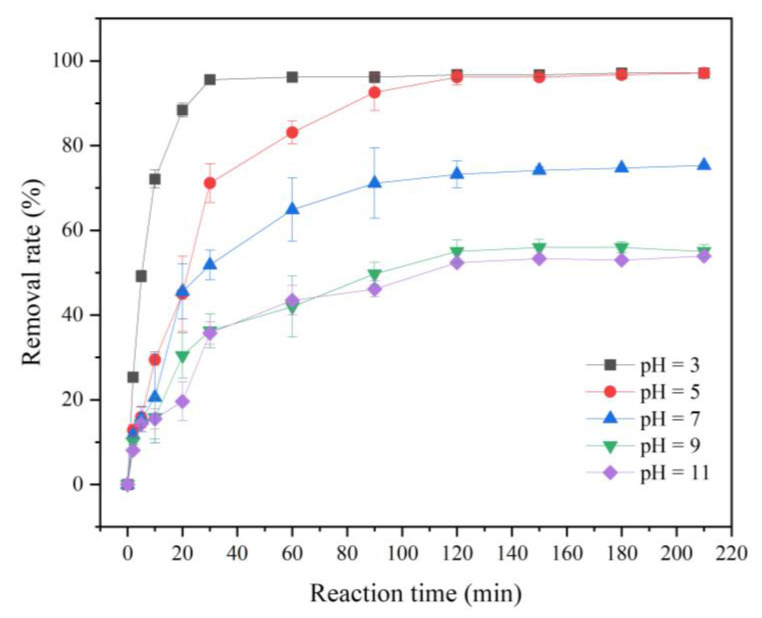
Removal rate of Cr(VI) at different reaction pH levels.

Under the condition of 25 °C and initial concentration of Cr(VI) of 20 mg/L, the effects of different pH levels (3, 5, 7, 9, 11) on the removal effect of Cr(VI) by FP-nZVI prepared with 0.8 mol/L ferric chloride hexahydrate solution were investigated. As shown in Figure 10, the reaction pH had a significant impact on the removal rate of Cr(VI). The lower the pH, the faster the reaction rate and the better the removal effect. The removal mainly occurred within 2 h. When the reaction time was 60 min, the removal rate of Cr(VI) was as high as 96% when pH = 3, but the rates were only 42% and 43% when pH = 9 and 11. This showed that the pH of the solution affected the redox reaction between nZVI and Cr(VI) [54] and that acidic conditions were more favorable for the removal of Cr(VI). This was because under acidic conditions, HCrO_4_^−^ was the main form of Cr(VI) [55], and there was more H^+^. On the one hand, the increase in H^+^ can accelerate the dissolution of nZVI, make nZVI release more reductive electrons, and accelerate the conversion of more Cr(VI) to the low-valence state. On the other hand, with the decrease in pH, the positive net charges of the adsorbent increase, and the electrostatic attraction between the adsorbent surface and the chromium oxyanions increases, which is conducive to the adsorption of Cr(VI) [56]. Therefore, the lower the pH was, the faster the reaction rate was and the more Cr(VI) was removed. In contrast, in alkaline environments, the nZVI surface made it easy to produce iron oxide/hydroxide precipitates, which would produce a passivating effect [10] and hinder the electron transfer of nZVI, resulting in a decrease in the reaction rate and thus inhibiting the reaction process. In addition, CrO_4_^2−^ was the main form of Cr(VI) in the alkaline environment, and the competition between high concentration of OH^−^ and chromium oxyanions for sorption sites was intensified [57], which further reduced the removal of Cr(VI). This was consistent with most literature reporting that the adsorption and reduction of Cr(VI) removal decreased with increasing pH [58,59].

#### 3.2.5. Effect of Aging on Cr(VI) Removal

The effect of natural oxidation of FP-nZVI on the removal of Cr(VI) was investigated. Six pieces of FP-nZVI prepared with 0.8 mol/L ferric chloride hexahydrate solution were placed in the same clean and open environment for different time periods (0, 7, 14, 21, 28, and 35 days) to oxidize naturally in air. Six pieces of FP-nZVI were immersed in six triangular flasks, each filled with 200 mL of a Cr(VI) solution of 20 mg/L. The pH = 5 was adjusted, the flasks were sealed with sealing film, and the temperature was controlled at 25 °C. The concentration of Cr(VI) was determined after shaking in a shaker and taking samples. The sampling scheme and testing method were the same as batch experiment 1.

**Figure 11:**
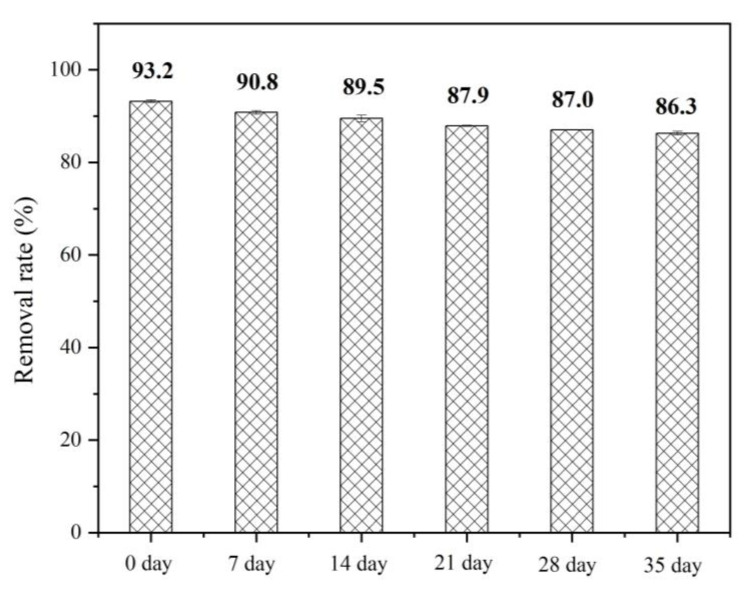
Removal effect of aging FP-nZVI on Cr(VI).

As shown in Figure 11, the removal rate of Cr(VI) decreased with increasing aging time. The main reason was that the formation of the oxide layer on the surface of nZVI hindered the electron transfer. However, the removal rate still remained above 85%, indicating that the FP-nZVI prepared in this study had good oxidation resistance in air. Under the condition of Cr(VI) concentration of 10 mg/L and pH = 5, the removal rates of Cr(VI) by CaCO_3_-coated nZVI prepared by Cheng et al. were 83.2% and 82.6%, respectively, when it was aged in air for 2 days and 30 days [60], which were lower than the removal rate (86.3%) of Cr(VI) by FP-nZVI aged for 30 days in this study under the condition of Cr(VI) concentration of 20 mg/L and pH = 5. This further showed that the FP-nZVI prepared in this study had good oxidation resistance.

#### 3.2.6. Kinetic Analysis

**Figure 12:**
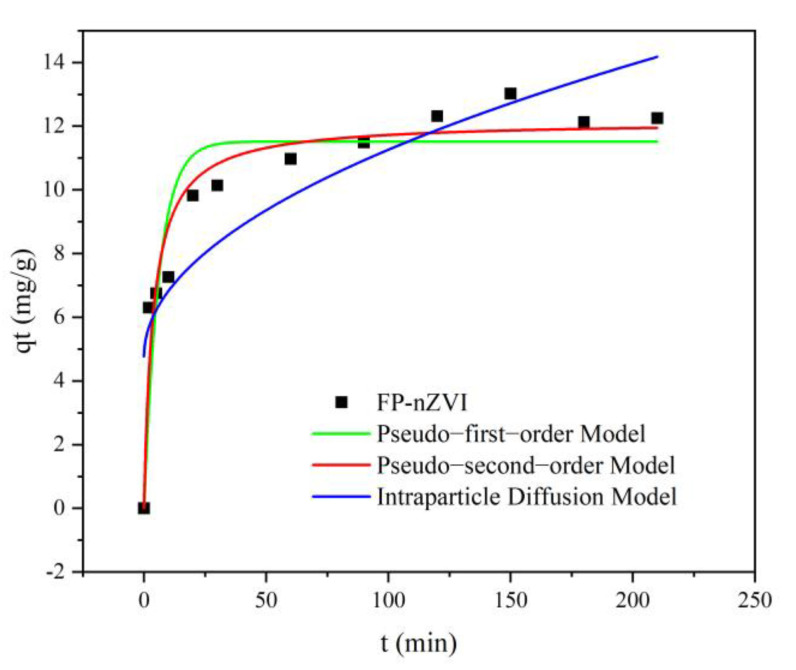
Kinetic model of Cr(VI) removal by FP-nZVI.

In order to understand the removal process of Cr(VI) by FP-nZVI, under the condition of 25 °C, pH=5, and Cr(VI) concentration of 20 mg/L, the kinetic experiment on Cr(VI) was carried out with FP-nZVI prepared from 0.8 mol/L ferric chloride hexahydrate solution. Pseudo-first-order and pseudo-second-order kinetic models and an intraparticle diffusion model were used to fit the experimental data. The pseudo-first-order and pseudo-second-order kinetic models and intraparticle diffusion model could be described by Equations (2)–(4):

(2) $$q_{t}=q_{e}\left(1-e^{-k_{1}t}\right)$$

(3) $$q_{t}=\frac{q_{e}^{2}k_{2}t}{1+q_{e}k_{2}t}$$

(4) $$q_{t}=k_{3}t^{0.5}+c$$

where *q_t_* (mg/g) and *q_e_* (mg/g) are the adsorption capacities at time t and equilibrium, respectively; *k*_1_ (min^−1^) is the pseudo-first-order kinetic rate constant; *k*_2_ (g·mg^−1^·min^−1^) is the pseudo-second-order kinetic rate constant; *k*_3_ (g·mg^−1^·min^−0.5^) is the rate constant of the intraparticle diffusion; and c is a constant. The fitted results and the parameters are shown in Figure 12 and Table 2. The R^2^ value of the pseudo-second-order kinetic model was 0.9386, which was greater than that of the pseudo-first-order kinetic model (0.8636) and the intraparticle diffusion model (0.7470). The pseudo-second-order kinetic model fitted the experimental data more accurately than the pseudo-first-order kinetic model and the intraparticle diffusion model, indicating that the adsorption process was mainly chemisorption. This was consistent with the results of Cr(VI) removal by amino-modified biochar loaded with nZVI [61].

#### 3.2.7. Isothermal Adsorption Analysis

**Figure 13:**
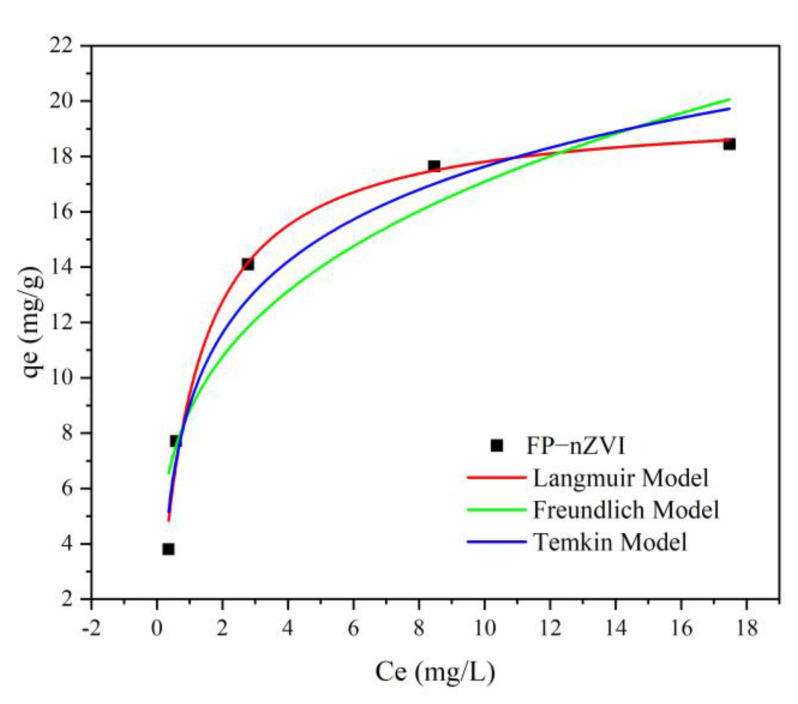
Fitting curve of Cr(VI) adsorption by FP-nZVI.

Figure 13 shows the fitting curves of the isothermal model of FP-nZVI and Cr(VI), and the correlation parameters are listed in Table 3. The correlation coefficient R^2^ of the Langmuir model was 0.9886, which was better than the coefficients 0.9635 of the Temkin model and 0.8944 of the Freundlich model. Therefore, the adsorption of Cr(VI) by FP-nZVI was more consistent with the Langmuir model, indicating that the adsorption of Cr(VI) by FP-nZVI was mainly based on monolayer chemisorption and that adsorption occurred on specific and homogeneous adsorption sites [62]. The maximum adsorption capacity obtained from the Langmuir model was estimated to be 19.7619 mg Cr g^−1^ FP-nZVI. Shu et al. used biochar-loaded nZVI prepared from almond shell for Cr(VI) removal and found that the isothermal data were consistent with the Langmuir model [63]. The comparison of adsorption capacity with other nZVI-based adsorbents is shown in Table S4 [17,19,42,58,64,65,66,67]. In addition, this study also investigated the adsorption of Cr(VI) by quartz sand and performed isothermal adsorption analysis to obtain parameters as the basis for the numerical simulations below. The results show that the adsorption of Cr(VI) by quartz sand was weak. The specific process of Cr(VI) adsorption study by quartz sand is shown in Text S4. An adsorption fitting diagram and related parameters are shown in Figure S1 and Table S5.

#### 3.2.8. Thermodynamic Analysis

The thermodynamic parameters were calculated by Equations (5)–(7):

(5) $$K_{d}=\frac{Q_{e}}{C_{e}}$$

(6) $$lnK_{d}=-\frac{\Delta H^{0}}{RT}+\frac{\Delta S^{0}}{R}$$

(7) $$\Delta G^{0}=\Delta H^{0}-T\Delta S^{0}$$

where *Q_e_* (mg/g) is the adsorption capacity at equilibrium, *C_e_* (mg/L) is the concentration of Cr(VI) at equilibrium, *K_d_* is the adsorption equilibrium constant, T (K) is temperature, R (8.314 J/mol/K) is the gas constant, enthalpies are represented by $\Delta H^{0}$ (KJ/mol), entropy change by $\Delta S^{0}$ (J/mol/K), and Gibbs free energy by $\Delta G^{0}$ (KJ/mol).

**Figure 14:**
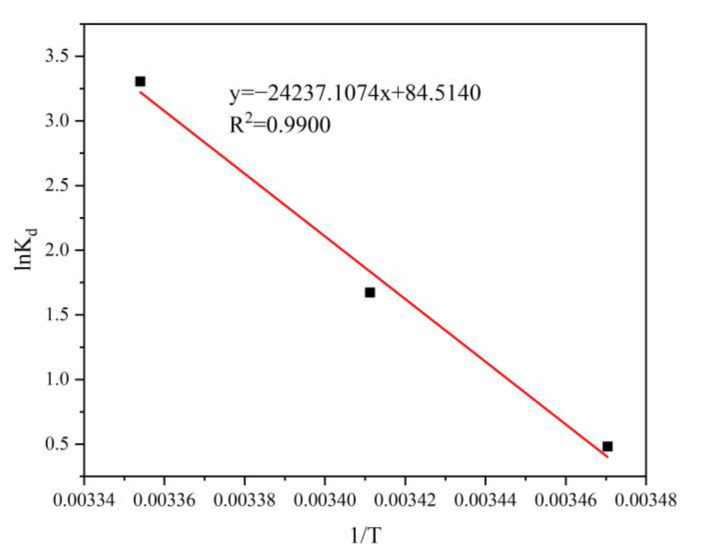
Fit line between lnK_d_ and 1/T.

The linear curve was fitted using 1/T versus lnK_d_, as shown in Figure 14. The corresponding thermodynamic parameters are shown in Table 4. The $\Delta H^{0}$ of Cr(VI) adsorption by FP-nZVI was positive, demonstrating an endothermic adsorption mechanism. $\Delta S^{0}$ was positive, indicating that during the adsorption process, the level of freedom in the procedures as well as the instability of the solid–liquid boundary increase [68]. $\Delta G^{0}$ below zero indicated that there was a spontaneous adsorption mechanism. In addition, when the adsorption temperature went up, the absolute value of $\Delta G^{0}$ grew, suggesting an increase in the degree of spontaneous Cr(VI) adsorption by FP-nZVI.

### 3.3. Removal Mechanism

**Figure 15:**
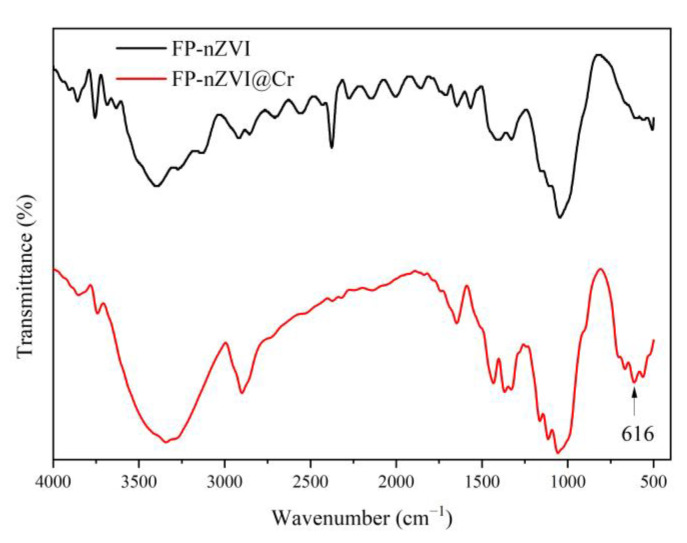
FTIR spectra of FP-nZVI and FP-nZVI@Cr.

**Figure 16:**
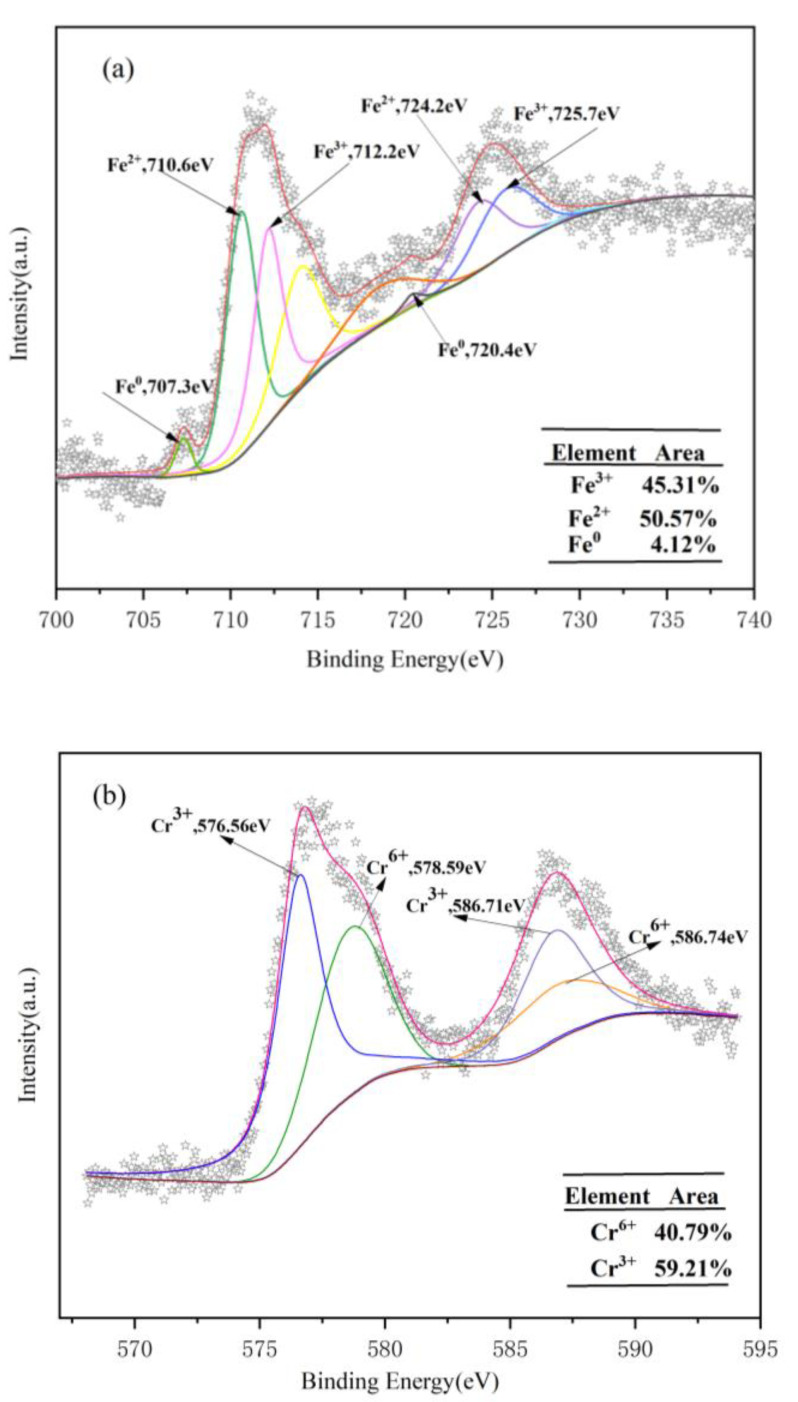
(**a**) Fe2p narrow-spectrum scan of FP-nZVI@Cr; (**b**) Cr2p narrow-spectrum scan of FP-nZVI@Cr.The lines with different colors are the fitting curves of the intensity of the corresponding valence states of the iron element or chromium element. The star curve is the fitting curve of the total strength curve of iron element or chromium element.

FP-nZVI prepared with 0.8 mol/L ferric chloride hexahydrate solution was used to remove Cr(VI) at 25 °C, pH = 5, and an initial concentration of Cr(VI) of 20 mg/L. The FTIR analysis of the post-reaction material (FP-nZVI@Cr) is shown in Figure 15. It can be seen from Figure 15 that a new absorption peak appeared at 616 cm^−1^, which may be attributed to the complexation effect of Fe and Cr during the coprecipitation process [37,69]. The surface element composition and chemical valence state of FP-nZVI@Cr were studied by XPS. The lines with different colors are the fitting curves of the intensity of the corresponding valence states of the iron element or chromium element. The star curve is the fitting curve of the total strength curve of iron element or chromium element. It can be seen from Figure 16a that after the removal reaction of Cr(VI) by FP-nZVI, the peak area of Fe^0^ decreased significantly from 12.98% to 4.12% and Fe(III) increased from 33.35% to 45.31%, indicating that Fe^0^ and Fe(II) were oxidized in the Cr(VI) removal process. Furthermore, as shown in Figure 16b, the binding energies of 578.59 eV and 586.74 eV are the characteristic peaks of Cr2p_3/2_ and Cr2p_1/2_ of Cr(VI), and the binding energies of 576.56 eV and 586.71 eV are the characteristic peaks of Cr2p_3/2_ and Cr2p_1/2_ of Cr(III). The peak areas of Cr(VI) and Cr(III) were estimated semi-quantitatively, indicating that 40.79% was in the Cr(VI) state, while about 59.21% was in the Cr(III) oxidation state. The existence of Cr(VI) indicated adsorption between FP-nZVI and Cr(VI), which may be due to the electrostatic interaction between FP-nZVI and Cr(VI) [57,70]. The presence of Cr(III) indicated that part of the adsorbed Cr(VI) was reduced to Cr(III). Similar results have been found in other research [71].

**Figure 17:**
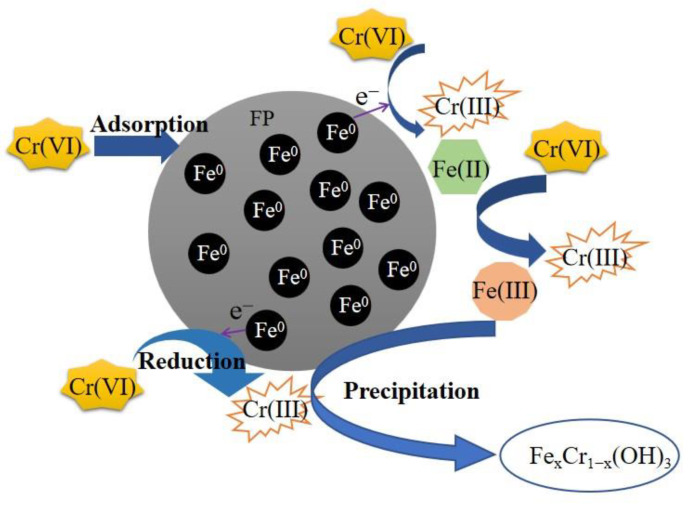
Removal mechanism of Cr(VI).

Therefore, the possible mechanisms for Cr(VI) removal can be inferred from the analysis results: (1) mass transfer of Cr(VI) occurred from bulk solution to the surface of FP-nZVI, and it was adsorbed; (2) then, Cr(VI) was reduced to Cr(III) by Fe^0^ or Fe(II), resulting in Fe^0^ being oxidized to Fe(II), and then Fe(II) continued to participate in the reduction reaction and eventually oxidized to Fe(III); (3) Fe(III) and Cr(III) were coprecipitated in the form of Fe_x_Cr_1−x_(OH)_3_ [72,73]. The removal mechanism is shown in Figure 17. The main reaction equations are shown in Equations (8)–(13) [41]:

(8) $$2{\mathrm{HCrO}}_{4}^{-}+3{\mathrm{Fe}}^{0}+14H^{+}\to 3{\mathrm{Fe}}^{2+}+2{\mathrm{Cr}}^{3+}+8H_{2}O$$

(9) $${\mathrm{HCrO}}_{4}^{-}+3{\mathrm{Fe}}^{2+}+7H^{+}\to 3{\mathrm{Fe}}^{3+}+{\mathrm{Cr}}^{3+}+4H_{2}O$$

(10) $${\mathrm{Cr}}_{2}O_{7}^{2-}+3{\mathrm{Fe}}^{0}+14H^{+}\to 3{\mathrm{Fe}}^{2+}+2{\mathrm{Cr}}^{3+}+7H_{2}O$$

(11) $${\mathrm{Cr}}_{2}O_{7}^{2-}+6{\mathrm{Fe}}^{2+}+14H^{+}\to 6{\mathrm{Fe}}^{3+}+2{\mathrm{Cr}}^{3+}+7H_{2}O$$

(12) $${\mathrm{xFe}}^{3+}+\left(1-x\right){\mathrm{Cr}}^{3+}+3H_{2}O\to {\mathrm{Fe}}_{x}{\mathrm{Cr}}_{1-x}{\left(\mathrm{OH}\right)}_{3}↓+3H^{+}$$

(13) $$6{\mathrm{Fe}}^{2+}+{\mathrm{Cr}}_{2}O_{7}^{2-}+7H_{2}O\to 6{\mathrm{Fe}}^{3+}+2\mathrm{Cr}{\left(\mathrm{OH}\right)}_{3}↓+8{\mathrm{OH}}^{-}$$

### 3.4. Column Experiments

**Figure 18:**
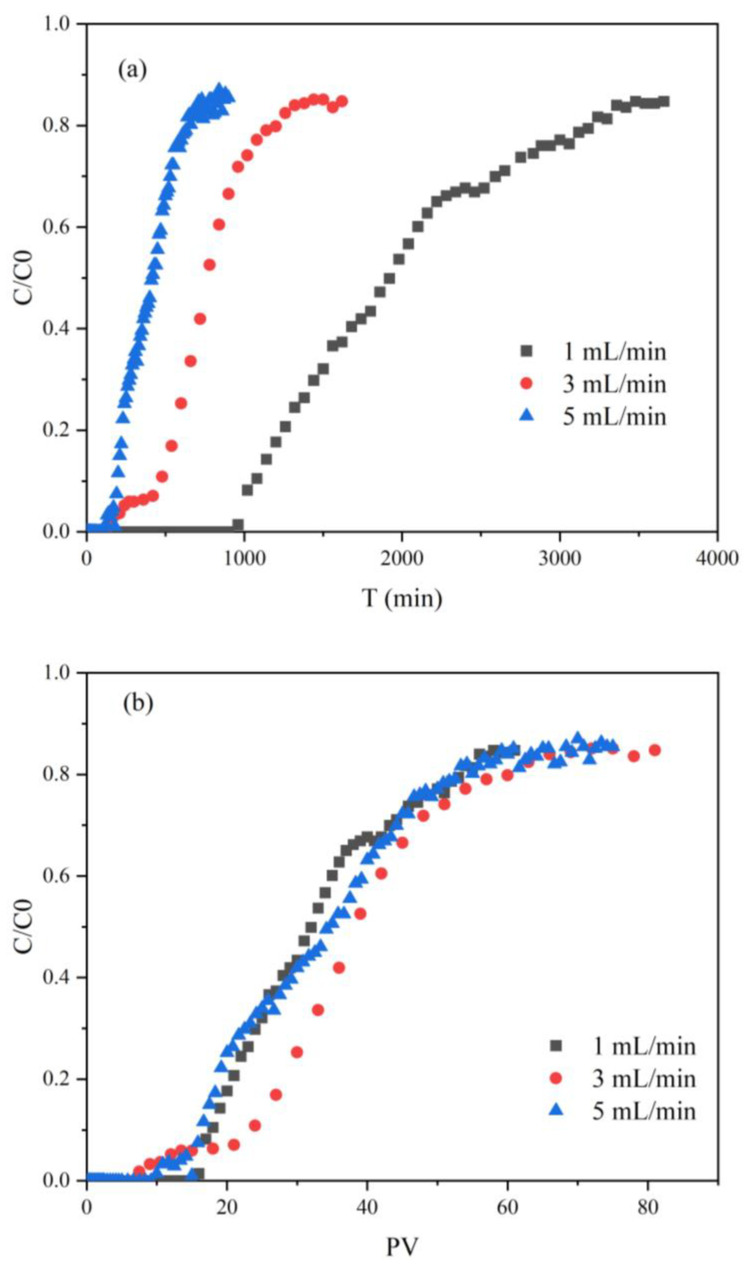
Removal effect of Cr(VI) in column experiment.(**a**) C/C_0_-T; (**b**) C/C_0_-PV.

Using ae breakthrough curve is a typical method to assess the ability of an adsorbent to remove contaminants [74]. The experimental column was a sand column with FP-nZVI interlayer, which represented the reaction barrier. A Cr(VI) solution with an initial concentration of 20 mg/L was pumped into the sand column at three flow rates (1, 3, and 5 mL/min). The outlet Cr(VI) concentration was measured according to the time interval. The variation curves of Cr(VI) concentration in the effluent with time and PV (pore volume) were plotted, as shown in Figure 18. When the flow rates were 1, 3, and 5 mL/min, the breakthrough times were 960, 150, and 120 min, respectively. With the increase in flow rate, the breakthrough time was shortened and the curve became steeper. This was because the larger the injection velocity, the stronger the convective dispersion effect and the larger the mass of Cr(VI) outflow per unit time. At the same time, the interaction time between Cr(VI) and FP-nZVI became shorter, which led to a faster increase in Cr(VI) concentration in the effluent and a steeper curve. This was consistent with the results of previous studies [75]. However, from the perspective of PV, the start of Cr(VI) breakthrough was related to the volume of Cr(VI) solution passed through. At the three flow rates, although the start of breakthrough time was different, all curves started to breakthrough at about 16 PV and reached the dynamic equilibrium state at approximately 60 PV, and the removal effect was basically the same. This indicated that the flow rate had an effect on the breakthrough time, which had a slight effect on the overall treatment effect. PV was the main factor affecting the equilibrium state and removal effect.

With an integral of breakthrough curve of C/C_0_-T or C/C_0_-PV, the capture capacity caused by reduction and adsorption could be calculated. The capture capacities at equilibrium time were 13.50, 16.46, and 14.45 mg Cr(VI) g^−1^ FP-nZVI at the flow rates of 1, 3, and 5 mL/min, respectively. In the column experiment, the maximum retention rate of Cr(VI) by the sand column during the period from the time of injection at the inlet to the time when the Cr(VI) concentration in the effluent was basically stable was calculated by Equations (14) and (15):

(14) $$M_{capture}=M_{total\;in}-M_{total\;out}$$

(15) $$Rate_{capture}=M_{capture}/M_{total\;in}$$

where *Rate_capture_* is the maximum retention rate of Cr(VI) by the sand column; *M_capture_* (mg) is the total mass of Cr(VI) retained by the sand column; *M_total in_* (mg) is the total mass of Cr(VI) flowing in from the inlet of the sand column; and *M_total out_* (mg) is the total mass of Cr(VI) flowing out from the outlet of the sand column.

The overall maximum retention rates of Cr(VI) in the sand column were 57.91%, 53.15%, and 50.39% at the flow rates of 1, 3, and 5 mL/min, respectively. The retention rate of Cr(VI) by the column decreased slightly with the increase in flow rate. Digiacomo et al. used sulphate green rust and S-nZVI to remove Cr(VI) in the column and found that the flow rate had a lower impact on the removal rate, but the higher the flow rate, the lower the removal rate [76]. This was consistent with the results of this study. The main reason for the decrease in the retention rate was that the larger the flow rate, the shorter the contact reaction time between Cr(VI) and FP-nZVI, and the reaction was not sufficient. With the injection of Cr(VI), the remaining active sites of FP-nZVI continued to decrease, and the Cr(VI) consumed by the reaction gradually decreased. Therefore, the Cr(VI) concentration in the effluent gradually increased.

### 3.5. Reactive Transport Model of Cr(VI) in Column

To further verify the reactive transport of Cr(VI), the breakthrough procedure was modeled using the CDE model in HYDRUS-1D. The water flow model was described by the modified Richards equations [77], given as follows in Equations (16) and (17):

(16) $$\frac{\partial \theta }{\partial t}=\frac{\partial }{\partial x}\left[K\left(\frac{\partial h}{\partial x}+\cos\alpha \right)\right]-S$$

(17) $$K\left(h,x\right)=K_{s}\left(x\right)K_{r}\left(h,x\right)$$

where *h* (cm) is the pressure head, θ is the volume water content, *t* (min) is the time, and *S* (min^−1^) is the source sink term. α is the angle between the flow direction and the vertical direction, and in this study, $\alpha =0^{{}^{\circ}}$. *x* (cm) is the vertical coordinate, and upward direction is defined as positive. *K* (cm/min) is the unsaturated hydraulic conductivity, *K_r_* (cm/min) is the relative hydraulic conductivity, and *K_s_* (cm/min) is the saturated hydraulic conductivity. The water flow was stable, and the composite porous media was saturated. The lower boundary was assumed as the constant flow boundary, and the upper boundary was the given head boundary. The water quality transport model considered two-site adsorption. The two-site model is a chemical non-equilibrium model, in which exchange sites are divided into two types. Type 1 sites (*S*_1_) are in equilibrium with the solution phase, and on type 2 sites (*S*_2_), sorption is considered to be time-dependent. In this study, sorption is described by the Langmuir equation, and the two-site model is given as Equation (18) [78]:

(18) $$\left(1+\frac{f\cdot \rho }{\theta }\left[\frac{b\cdot K_{L}}{{\left(1+K_{L}\cdot c\right)}^{2}}\right]\right)\cdot \frac{\partial c}{\partial t}=D\frac{\partial^{2}c}{\partial x^{2}}-v\frac{\partial c}{\partial x}-\frac{\alpha \cdot \rho }{\theta }\cdot \left[\left(1-f\right)\cdot \frac{b\cdot K_{L}\cdot c}{1+K_{L}\cdot c}-S_{2}\right]$$

where f is the equilibrium site fraction, α (min^−1^) is the first-order kinetic rate coefficient, *K*_L_ (mL/g) is the Langmuir adsorption coefficient, and *b* (mg/kg) is the maximum adsorption capacity of the medium to solute. *S*_2_ is the solid phase concentration at the type 2 site, and θ is the volumetric water content. c (mg/mL) is the solute concentration in the liquid phase, *D* (cm^2^/min) is the dispersion coefficient, *v* (cm/min) is pore water velocity, *x* (cm) is the spatial position, ρ (g/cm^−3^) is the bulk density of porous media, and t is time. HYDRUS-1D requires input parameters such as $q\left(q\;=\frac{Q}{A}\right)$, θ, $\lambda \left(\lambda \;=\frac{D}{v}\right),\;b$, $K_{L},\;f,\;$and α to build a nonlinear non-equilibrium model (NLNE). In the water quality transport model, the solute concentration at each position of the column was assumed as 0 at the initial moment. The lower boundary was the given concentration boundary, and the upper boundary was assumed as the flux boundary.

It had been found that sorption parameters obtained from static batch experiments may not be suitable for determining solute transport in column experiments [79]. Because the interaction between FP-nZVI and Cr(VI) under static conditions was confined to a relatively stable experimental environment, however, the soil solution system composed of soil particles and pore solutions under dynamic conditions would have inhomogeneity, which could affect the chemical reaction process of heavy metal ions in soil. Therefore, the reaction state and parameters expressed in static experiments cannot fully reflect the mass transfer processes such as sorption, reaction conditions, and transport state in the soil [80]. *K*_L_ and *b* obtained from static batch experiments can be substituted into the simulation model as initial parameters. The calculated values were fitted with the observed breakthrough data and curves of the column experiments for iterative adjustment, and thus, the parameters *K*_L_ and *b* were trimmed [81]. The kinetic parameters (f and α) were obtained automatically by the inverse solution module of HYDRUS-1D, and the model parameters are shown in Table 5. The decrease in f indicated that more equilibrium adsorption was transformed into non-equilibrium adsorption. α described the adsorption/desorption ratio of Cr(VI) in time.

**Figure 19:**
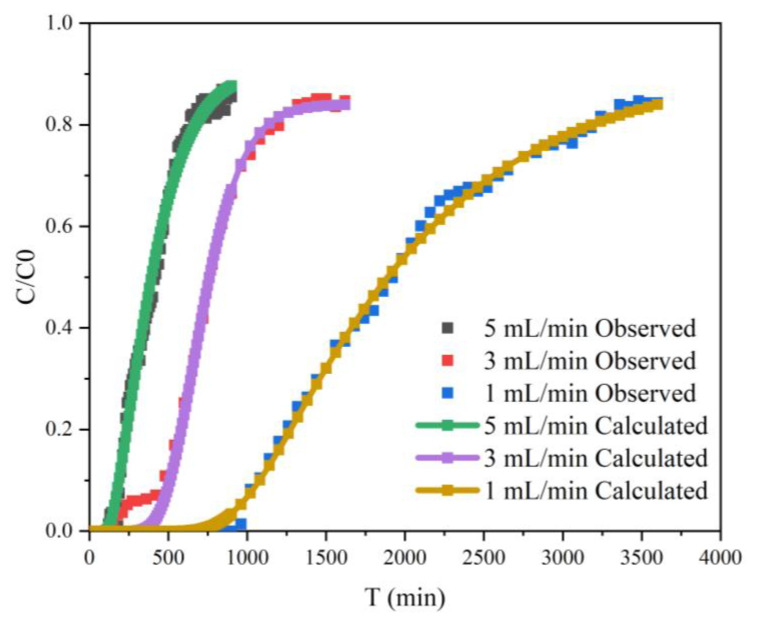
Fitting of observed and calculated values of Cr(VI) concentration in effluent at three flow rates.

The comparison between the observed breakthrough curves and calculated values of Cr(VI) at three flow rates is shown in Figure 19. The overall changing trend of the calculated and observed values was consistent. The root mean square error (RMSE) ranged from 0.489% to 1.204%, and R^2^ values were all greater than 0.99. This indicated that the two-site model of HYDRUS-1D can simulate well the transport of Cr(VI) in composite porous media. Montalvo et al. used the two-site model to fit the reactive transport of arsenate in porous media, and its kinetic parameters were also obtained by inversion. The fitted breakthrough curve could match the observed data [78]. The model was helpful to understand the transport mechanism of Cr(VI) in porous media. In this study, both K_L_ and b after adjustment were smaller than those of batch experiments. This was because, in the static experimental environment, FP-nZVI can provide more reaction sites, and the reaction time of FP-nZVI and Cr(VI) was more sufficient, but column experimental conditions did not have these characteristics. At the end of the column experiment, C/C_0_ = 0.8, and there still existed a retaining effect on nZVI in the column, which had not reacted completely and not reached the adsorption capacity of FP-nZVI. This was one of the reasons why the adsorption parameters of the column experiment were smaller than those of the static batch experiment. In addition, the K_L_ and b obtained from the inversion can provide some basis for the prediction of the transport of Cr(VI) in groundwater.

## 4. Conclusions

Batch experiments showed that the removal rate of Cr(VI) increased with the increase in iron loading and reaction temperature, and acidic conditions were more conducive to the removal of Cr(VI) by FP-nZVI. The removal rate was higher than 86% at 35 days of aging, indicating that FP-nZVI had very good oxidation resistance. The removal of Cr(VI) by FP-nZVI conformed to the pseudo-second-order kinetic model, and FP-nZVI had a good adsorption capacity for Cr(VI), with the maximum adsorption capacity of b = 19.7619 mg Cr g^−1^ by FP-nZVI. The removal process of Cr(VI) by FP-nZVI included adsorption, reduction, and coprecipitation. Column experiments discovered that increasing flow rate slightly decreased the removal rate of Cr(VI) and pore volume ratio of fluid passing through was the main factor affecting the removal rate of Cr(VI). HYDRUS-1D simulation results show that the two-site model can simulate well the transport of Cr(VI) in composite porous media. The inversion parameters provided a basis for the transport of Cr(VI) in groundwater. This study demonstrated that FP-nZVI had a strong ability for removal of Cr(VI) and has potential practical application value in the remediation of heavy-metal-contaminated groundwater.

## Figures and Tables

**Table 1:** Operating conditions.

Batch Experiment	Iron Concentration (mol/L)	Initial Concentration of Cr(VI) (mg/L)	Temperature (°C)	pH
1	0.1, 0.2, 0.5, 0.8, 1.0	20	25	5
2	0.8	5, 10, 20, 30, 40	25	5
3	0.8	20	15, 20, 25, 30, 35	5
4	0.8	20	25	3, 5, 7, 9, 11

**Table 2:** Kinetic model parameters; standard error is in parentheses.

Kinetic Models	Parameters			
	*k*	*q_e_* (mg/g)	*c*	R^2^
Pseudo-first order	0.1634 (0.0410)	11.52 (0.5323)	/	0.8636
Pseudo-second order	0.0220 (0.0057)	12.16 (0.4290)	/	0.9386
Intraparticle diffusion	0.6492 (0.1195)	/	4.7668 (1.0213)	0.7470

**Table 3:** Isothermal model parameters; standard error is in parentheses.

Medium	Langmuir Equation			Freundlich Equation			Temkin Equation		
	K_L_ (L/mg)	b (mg/g)	R^2^	K_F_ (L/g)	n	R^2^	K_T_ (L/mg)	b (KJ/mol)	R^2^
FP-nZVI	0.9095 (0.1383)	19.7619 (0.7321)	0.9886	8.8097 (1.3895)	3.4771 (0.0692)	0.8944	11.1043 (4.5292)	3.7433 (0.4207)	0.9635

**Table 4:** Thermodynamic parameters; standard error is in parentheses.

$\Delta H^{0}\;(\mathrm{KJ}/\mathrm{mol})$	$\Delta S^{0}\;(J/\mathrm{mol}/K)$	$\Delta G^{0}\;(\mathrm{KJ}/\mathrm{mol})$		
		288.15K	293.15K	298.15K
201.5073 (20.2468)	702.6494 (69.0864)	−0.9611	−4.4744	−7.9876

**Table 5:** Model parameters; the initial values are in parentheses.

Flow Rate (mL/min)	Medium	*D* (cm^2^/min)	θ	*K* (cm/min)	$K_{L}\;(L/\mathrm{mg})$	*b* (mg/g)	*f*	$\alpha \;(\min^{-1})$	RMSE	R^2^
1	Quartz sand	0.0978	0.5042	4.7181	0.0006003 (0.0038)	0.005792 (0.10062)	0.5030	0.5783	0.00489	0.99839
	FP-nZVI	0.1369	0.9908	11.0403	0.002002 (0.9095)	0.133217 (19.7619)	0.6553	0.001361		
3	Quartz sand	0.1744	0.5042	4.7181	0.0006003 (0.0038)	0.005792 (0.10062)	0.2057	0.9582	0.01204	0.99711
	FP-nZVI	0.2383	0.9908	11.0403	0.002002 (0.9095)	0.133217 (19.7619)	0.1125	0.5959		
5	Quartz sand	0.2369	0.5042	4.7181	0.0006003 (0.0038)	0.005792 (0.10062)	0.4556	0.01271	0.00638	0.99578
	FP-nZVI	0.2889	0.9908	11.0403	0.002002 (0.9095)	0.133217 (19.7619)	0.4475	0.01197		

## Data Availability

Not applicable.

## Supplementary Materials

The following supporting information can be downloaded at: https://www.mdpi.com/article/10.3390/ijerph20031867/s1. Text S1: Porosity experiment; Text S2: Permeability coefficient experiment; Text S3: Dispersion coefficient experiment; Text S4: Adsorption Experiment of Cr(VI) by quartz sand; Figure S1: Adsorption Fitting Curve of Cr(VI) by quartz sand; Table S1: Porosity-related parameters and results; Table S2: Permeability-coefficient-related parameters and results; Table S3: Dispersion-coefficient-related parameters and results; Table S4: Comparison of removal capacity for Cr(VI) by FP-nZVI with other adsorbents; Table S5. Isothermal model parameters of quartz sand to Cr(VI), standard error was in parentheses.

## References

[1] Nandi R., Laskar S., Saha B. (2016). Surfactant-promoted enhancement in bioremediation of hexavalent chromium to trivalent chromium by naturally occurring wall algae. Res. Chem. Intermed..

[2] Guo C., Han B.C.P. (2021). Optimization of Chromium(Ⅵ) Removal by Pseudomonas. J. Guangxi Norm. Univ..

[3] Ren Y., Han Y., Lei X., Lu C., Liu J., Zhang G., Zhang B., Zhang Q. (2020). A magnetic ion exchange resin with high efficiency of removing Cr (VI). Colloids Surf. A: Physicochem. Eng. Asp..

[4] Rivero M.J., Primo O., Ortiz M.I. (2004). Modelling of Cr(VI) removal from polluted groundwaters by ion exchange. J. Chem. Technol. Biotechnol..

[5] Roy Choudhury P., Majumdar S., Sahoo G.C., Saha S., Mondal P. (2018). High pressure ultrafiltration CuO/hydroxyethyl cellulose composite ceramic membrane for separation of Cr (VI) and Pb (II) from contaminated water. Chem. Eng. J..

[6] Yao Z., Du S., Zhang Y., Zhu B., Zhu L., John A.E. (2015). Positively charged membrane for removing low concentration Cr(VI) in ultrafiltration process. J. Water Process Eng..

[7] Wang S. (2022). Removal of Chromium Hexavalent from Water by Modification of PEG Stabilized Nano Zero-Valent Iron Composite. Master’s Thesis.

[8] Chen Y., An D., Sun S., Gao J., Qian L. (2018). Reduction and Removal of Chromium VI in Water by Powdered Activated Carbon. Materials.

[9] Dong H., Deng J., Xie Y., Zhang C., Jiang Z., Cheng Y., Hou K., Zeng G. (2017). Stabilization of nanoscale zero-valent iron (nZVI) with modified biochar for Cr(VI) removal from aqueous solution. J. Hazard. Mater..

[10] Li J., Fan M., Li M., Liu X. (2020). Cr(VI) removal from groundwater using double surfactant-modified nanoscale zero-valent iron (nZVI): Effects of materials in different status. Sci. Total Environ..

[11] Fang Y., Wen J., Zhang H., Wang Q., Hu X. (2020). Enhancing Cr(VI) reduction and immobilization by magnetic core-shell structured NZVI@MOF derivative hybrids. Environ. Pollut.

[12] Jing Q., You W., Qiao S., Ma Y., Ren Z. (2023). Comprehensive understanding of adsorption and reduction on 2,4-DCP and Cr(VI) removal process by NZVI-rGO: Performance and mechanism. J. Water Process Eng..

[13] Soliemanzadeh A., Fekri M. (2017). The application of green tea extract to prepare bentonite-supported nanoscale zero-valent iron and its performance on removal of Cr(VI): Effect of relative parameters and soil experiments. Microporous Mesoporous Mater..

[14] Han P., Xie J., Qin X., Yang X., Zhao Y. (2022). Experimental study on in situ remediation of Cr(VI) contaminated groundwater by sulfidated micron zero valent iron stabilized with xanthan gum. Sci Total Environ..

[15] Shi D., Ouyang Z., Zhao Y., Xiong J., Shi X. (2019). Catalytic Reduction of Hexavalent Chromium Using Iron/Palladium Bimetallic Nanoparticle-Assembled Filter Paper. Nanomaterials.

[16] Yu Q., Guo J., Muhammad Y., Li Q., Lu Z., Yun J., Liang Y. (2020). Mechanisms of enhanced hexavalent chromium removal from groundwater by sodium carboxymethyl cellulose stabilized zerovalent iron nanoparticles. J. Environ. Manag..

[17] Zhao R., Zhou Z., Zhao X., Jing G. (2019). Enhanced Cr(VI) removal from simulated electroplating rinse wastewater by amino-functionalized vermiculite-supported nanoscale zero-valent iron. Chemosphere.

[18] Bai X., Dong J., Wang M., Yuan Z., He L. (2015). Review on Remediation of Heavy Metal Contaminated Groundwater by Modified Nanoscale Zero-Valent Iron. Yellow River.

[19] Zhou H., Ma M., Zhao Y., Baig S.A., Hu S., Ye M., Wang J. (2022). Integrated green complexing agent and biochar modified nano zero-valent iron for hexavalent chromium removal: A characterisation and performance study. Sci. Total Environ..

[20] Liang W., Wang G., Peng C., Tan J., Wan J., Sun P., Li Q., Ji X., Zhang Q., Wu Y. (2022). Recent advances of carbon-based nano zero valent iron for heavy metals remediation in soil and water: A critical review. J. Hazard Mater..

[21] Tielong L., Bing G., Na Z., Zhaohui J., Xinhua Q. (2009). Hexavalent chromium removal from water using chitosan-Fe0nanoparticles. J. Phys. Conf. Ser..

[22] Asad M.A., Khan U.T., Krol M.M. (2021). Subsurface transport of carboxymethyl cellulose (CMC)-stabilized nanoscale zero valent iron (nZVI): Numerical and statistical analysis. J. Contam. Hydrol..

[23] Akmanova A., Han S., Lee W. (2021). Enhanced degradation of aqueous doxycycline in an aerobic suspension system with pretreated sucrose-modified nano-zero-valent iron. J. Environ. Chem. Eng..

[24] Li Q., Wang H., Chen Z., He X., Liu Y., Qiu M., Wang X. (2021). Adsorption-reduction strategy of U(VI) on NZVI-supported zeolite composites via batch, visual and XPS techniques. J. Mol. Liq..

[25] Jin X., Chen Z., Zhou R., Chen Z. (2015). Synthesis of kaolin supported nanoscale zero-valent iron and its degradation mechanism of Direct Fast Black G in aqueous solution. Mater. Res. Bull..

[26] Baldermann A., Kaufhold S., Dohrmann R., Baldermann C., Letofsky-Papst I., Dietzel M. (2021). A novel nZVI–bentonite nanocomposite to remove trichloroethene (TCE) from solution. Chemosphere.

[27] Xu M., Ma X., Chen Y., Hu L., Wang B., Qiu M. (2022). Spectroscopic investigation of Cr(VI) sorption on nZVI/biochar composites. J. Mol. Liq..

[28] Qu G., Kou L., Wang T., Liang D., Hu S. (2017). Evaluation of activated carbon fiber supported nanoscale zero-valent iron for chromium (VI) removal from groundwater in a permeable reactive column. J Environ. Manag..

[29] Liu F., Yang J., Zuo J., Ma D., Gan L., Xie B., Wang P., Yang B. (2014). Graphene-supported nanoscale zero-valent iron: Removal of phosphorus from aqueous solution and mechanistic study. J. Environ. Sci..

[30] Liu X., Zhang S., Zhang X., Guo H., Cao X., Lou Z., Zhang W., Wang C. (2022). A novel lignin hydrogel supported nZVI for efficient removal of Cr(VI). Chemosphere.

[31] Arshadi M., Abdolmaleki M.K., Eskandarloo H., Azizi M., Abbaspourrad A. (2018). Synthesis of Highly Monodispersed, Stable, and Spherical NZVI of 20–30 nm on Filter Paper for the Removal of Phosphate from Wastewater: Batch and Column Study. ACS Sustain. Chem. Eng..

[32] Yang Y. (2021). Preparation of Fe^0^ @ Cellulose Fiber Filter Material and Its Wastewater Treatment. Master’s Thesis.

[33] Yu P., Yu H., Sun Q., Ma B. (2019). Filter paper supported nZVI for continuous treatment of simulated dyeing wastewater. Sci. Rep..

[34] Kamal T., Khan S.B., Asiri A.M. (2016). Synthesis of zero-valent Cu nanoparticles in the chitosan coating layer on cellulose microfibers: Evaluation of azo dyes catalytic reduction. Cellulose.

[35] Lakkaboyana S.K., Khantong S., Asmel N.K., Obaidullah S., Kumar V., Kannan K., Venkateswarlu K., Yuzir A., Wan Yaacob W.Z. (2021). Indonesian Kaolin supported nZVI (IK-nZVI) used for the an efficient removal of Pb(II) from aqueous solutions: Kinetics, thermodynamics and mechanism. J. Environ. Chem. Eng..

[36] Lu S. (2017). Preparation and Properties of DMDAAC-Modified Cellulose Filter Paper. Master’s Thesis.

[37] Wang Y., Yu L., Wang R., Wang Y., Zhang X. (2020). A novel cellulose hydrogel coating with nanoscale Fe(0) for Cr(VI) adsorption and reduction. Sci Total Environ..

[38] Sun Y.P., Li X.Q., Cao J., Zhang W.X., Wang H.P. (2006). Characterization of zero-valent iron nanoparticles. Adv. Colloid Interface Sci..

[39] Wang X., Wang T., Ma J., Liu H., Ning P. (2018). Synthesis and characterization of a new hydrophilic boehmite-PVB/PVDF blended membrane supported nano zero-valent iron for removal of Cr(VI). Sep. Purif. Technol..

[40] Duchemin B., Le Corre D., Leray N., Dufresne A., Staiger M.P. (2015). All-cellulose composites based on microfibrillated cellulose and filter paper via a NaOH-urea solvent system. Cellulose.

[41] Wang Z., Chen G., Wang X., Li S., Liu Y., Yang G. (2020). Removal of hexavalent chromium by bentonite supported organosolv lignin-stabilized zero-valent iron nanoparticles from wastewater. J. Clean. Prod..

[42] Huang X., Niu X., Zhang D., Li X., Li H., Wang Z., Lin Z., Fu M. (2022). Fate and mechanistic insights into nanoscale zerovalent iron (nZVI) activation of sludge derived biochar reacted with Cr(VI). J. Environ. Manag..

[43] Eljamal R., Eljamal O., Maamoun I., Yilmaz G., Sugihara Y. (2020). Enhancing the characteristics and reactivity of nZVI: Polymers effect and mechanisms. J. Mol. Liq..

[44] Luo Z., Sheng K., Yin K. (2022). Distinctive aging and inhibiting effects of microplastics between fresh and sulfidated nano-zero valent iron for various metal adsorption. Chem. Eng. J..

[45] Wu Y.H.S. (2021). Ultrasonic Assisted Synthesis of Spherical and Irregular Cubic Titanium Dioxide Particles. Chem. Manag..

[46] Zheng Y., Zhang X., Wu M., Liu Y., Zhan J. (2022). Enhanced selective nitrate-to-nitrogen reduction by aerosol-assisted iron-carbon composites: Insights into the key factors. Chemosphere.

[47] Lin Y.H., Tseng H.H., Wey M.Y., Lin M.D. (2010). Characteristics of two types of stabilized nano zero-valent iron and transport in porous media. Sci Total Environ..

[48] Zhou X., Lv B., Zhou Z., Li W., Jing G. (2015). Evaluation of highly active nanoscale zero-valent iron coupled with ultrasound for chromium(VI) removal. Chem. Eng. J..

[49] Zhang S., Wang T., Guo X., Chen S., Wang L. (2022). Adsorption and reduction of trichloroethylene by sulfidated nanoscale zerovalent iron (S-nZVI) supported by Mg(OH)_2_. Environ. Sci. Pollut Res. Int..

[50] Zhang Y., Ma X. (2018). Removal of chromium(VI) from water by modified nano zero-valent iron. Appl. Chem. Ind..

[51] Fazlzadeh M., Rahmani K., Zarei A., Abdoallahzadeh H., Nasiri F., Khosravi R. (2017). A novel green synthesis of zero valent iron nanoparticles (NZVI) using three plant extracts and their efficient application for removal of Cr(VI) from aqueous solutions. Adv. Powder Technol..

[52] Wang Y., Gong Y., Lin N., Jiang H., Wei X., Liu N., Zhang X. (2022). Cellulose hydrogel coated nanometer zero-valent iron intercalated montmorillonite (CH-MMT-nFe0) for enhanced reductive removal of Cr(VI): Characterization, performance, and mechanisms. J. Mol. Liq..

[53] Mao Y. (2011). Treatment of Hexavalent Chromium in Groundwater Using Zero-Valent Iron Nanoparticles. Master’s Thesis.

[54] Kantar C., Ari C., Keskin S., Dogaroglu Z.G., Karadeniz A., Alten A. (2015). Cr(VI) removal from aqueous systems using pyrite as the reducing agent: Batch, spectroscopic and column experiments. J. Contam. Hydrol..

[55] Xu H., Gao M., Hu X., Chen Y., Li Y., Xu X., Zhang R., Yang X., Tang C., Hu X. (2021). A novel preparation of S-nZVI and its high efficient removal of Cr(VI) in aqueous solution. J. Hazard Mater..

[56] Gheju M., Balcu I., Mosoarca G. (2016). Removal of Cr(VI) from aqueous solutions by adsorption on MnO_2_. J. Hazard Mater..

[57] Fenti A., Chianese S., Iovino P., Musmarra D., Salvestrini S. (2020). Cr(VI) Sorption from Aqueous Solution: A Review. Appl. Sci..

[58] Wu L., Liao L., Lv G., Qin F. (2015). Stability and pH-independence of nano-zero-valent iron intercalated montmorillonite and its application on Cr(VI) removal. J. Contam. Hydrol..

[59] Kyzas G.Z., Kostoglou M., Lazaridis N.K. (2009). Copper and chromium(VI) removal by chitosan derivatives—Equilibrium and kinetic studies. Chem. Eng. J..

[60] Cheng Y., Dong H., Hao T. (2021). CaCO_3_ coated nanoscale zero-valent iron (nZVI) for the removal of chromium(VI) in aqueous solution. Sep. Purif. Technol..

[61] Hou S., Tian H., Huang C., Wang P., Zeng J., Peng H., Liu S., Li A. (2020). Removal of Cr(VI) from aqueous solution by amino-modified biochar supported nano zero-valent iron. Acta Sci. Circumstantiae.

[62] Li Q., Pan H., Yan W. (2022). Adsorption Isotherm and Kinetic of Peanut Shells Modified by Ionic Liquid for Cr(VI). Hydrometall. China.

[63] Shu Y., Ji B., Cui B., Shi Y., Wang J., Hu M., Luo S., Guo D. (2020). Almond Shell-Derived, Biochar-Supported, Nano-Zero-Valent Iron Composite for Aqueous Hexavalent Chromium Removal: Performance and Mechanisms. Nanomaterials.

[64] Mortazavian S., An H., Chun D., Moon J. (2018). Activated carbon impregnated by zero-valent iron nanoparticles (AC/nZVI) optimized for simultaneous adsorption and reduction of aqueous hexavalent chromium: Material characterizations and kinetic studies. Chem. Eng. J..

[65] Liu J., Mwamulima T., Wang Y., Fang Y., Song S., Peng C. (2017). Removal of Pb(II) and Cr(VI) from aqueous solutions using the fly ash-based adsorbent material-supported zero-valent iron. J. Mol. Liq..

[66] Li X., Ai L., Jiang J. (2016). Nanoscale zerovalent iron decorated on graphene nanosheets for Cr(VI) removal from aqueous solution: Surface corrosion retard induced the enhanced performance. Chem. Eng. J..

[67] Jia Z., Shu Y., Huang R., Liu J., Liu L. (2018). Enhanced reactivity of nZVI embedded into supermacroporous cryogels for highly efficient Cr(VI) and total Cr removal from aqueous solution. Chemosphere.

[68] Liu C., Lu J., Tan Y., Chen B., Yang P. (2022). Removal of U(VI) from wastewater by sulfhydryl-functionalized biomass carbon supported nano-zero-valent iron through synergistic effect of adsorption and reduction. Mater. Sci. Eng. B.

[69] Liu Q., Xu M., Li F., Wu T., Li Y. (2016). Rapid and effective removal of Cr(VI) from aqueous solutions using the FeCl3/NaBH4 system. Chem. Eng. J..

[70] Zhang S., Lyu H., Tang J., Song B., Zhen M., Liu X. (2019). A novel biochar supported CMC stabilized nano zero-valent iron composite for hexavalent chromium removal from water. Chemosphere.

[71] Geng B., Jin Z., Li T., Qi X. (2009). Preparation of chitosan-stabilized Fe(0) nanoparticles for removal of hexavalent chromium in water. Sci. Total Environ..

[72] Yang C., Ge C., Li X., Li L., Wang B., Lin A., Yang W. (2021). Does soluble starch improve the removal of Cr(VI) by nZVI loaded on biochar?. Ecotoxicol. Environ. Saf..

[73] Bian H., Wan J., Muhammad T., Wang G., Sang L., Jiang L., Wang H., Zhang Y., Peng C., Zhang W. (2021). Computational study and optimization experiment of nZVI modified by anionic and cationic polymer for Cr(VI) stabilization in soil: Kinetics and response surface methodology (RSM). Environ. Pollut.

[74] Saleh T.A., Al-Absi A.A. (2017). Kinetics, isotherms and thermodynamic evaluation of amine functionalized magnetic carbon for methyl red removal from aqueous solutions. J. Mol. Liq..

[75] Mondal M.K. (2009). Removal of Pb(II) ions from aqueous solution using activated tea waste: Adsorption on a fixed-bed column. J. Environ. Manag..

[76] Digiacomo F., Tobler D.J., Held T., Neumann T. (2020). Immobilization of Cr(VI) by sulphate green rust and sulphidized nanoscale zerovalent iron in sand media: Batch and column studies. Geochem. Trans..

[77] Jiang S., Pang L., Buchan G.D., Šimůnek J., Noonan M.J., Close M.E. (2010). Modeling water flow and bacterial transport in undisturbed lysimeters under irrigations of dairy shed effluent and water using HYDRUS-1D. Water Res..

[78] Montalvo D., Vanderschueren R., Fritzsche A., Meckenstock R.U., Smolders E. (2018). Efficient removal of arsenate from oxic contaminated water by colloidal humic acid-coated goethite: Batch and column experiments. J. Clean. Prod..

[79] Banzhaf S., Hebig K.H. (2016). Use of column experiments to investigate the fate of organic micropollutants—A review. Hydrol. Earth Syst. Sci..

[80] Li P. (2019). Study on the Effect of Nano-zero Valent Iron System on Removal and Filtration Characteristics and Transformation Mechanism of Cr(VI) in Soil. Ph.D. Thesis.

[81] Hanna K., Rusch B., Lassabatere L., Hofmann A., Humbert B. (2010). Reactive transport of gentisic acid in a hematite-coated sand column: Experimental study and modeling. Geochim. Et Cosmochim. Acta.

