# Supplementary material for: Removal of Hexavalent Chromium in Aqueous Solution by Cellulose Filter Paper Loaded with Nano-Zero-Valent Iron: Performance Investigation and Numerical Modeling
Source: Int J Environ Res Public Health. 2023 Jan 19;20(3):1867. doi: 10.3390/ijerph20031867 (PMC9915128; doi:10.3390/ijerph20031867)
Supplement: Supplementary file 1 [file ijerph-20-01867-s001.zip › ijerph-2176671-supplementary.pdf]

Supplementary Information

Removal of hexavalent chromium in aqueous solution by cellulose filter paper loaded with nano zero valent iron: performance investigation and numerical modeling

Submitted to

International Journal of Environmental Research and Public Health

Huali Li [1], Zhongyu Ren [1], Dan Huang [2] and Qi Jing [1] *, Haokai Tang [1]

[1] Institute of Water Resources and Engineering, Beijing University of Technology , Beijing 10024, China

[2] Songliao Water Conservancy Commission, Songliao Basin Water and Soil Conservation Monitoring Center Station, Changchun 130021, China

[*] Corresponding author: Tel.: (+86)18501950178; E-mail address: jingqi@bjut.edu.cn

Summary of contents:

Text S1-S4;

Figure S1;

Table S1-S5

Text S1. Porosity experiment.

The quartz sand (or FP-nZVI) with volume V_1 was filled in the plexiglas column (internal diameter = 3 cm, thickness = 0.3 cm, height = 23 cm), and then the deionized water was injected into the column from the bottom. After the quartz sand (or FP-nZVI) was saturated, the volume of the deionized water was recorded as V_2.

Porosity $n=V_2/V_1$

Text S2. Permeability coefficient experiment.

A plexiglas column (internal diameter = 3 cm, thickness = 0.3 cm, height = 23 cm) was filled with quartz sand (or FP-nZVI) of height L. The water surface height was maintained constant during the test. The amount of water (Q) flowing through the quartz sand (or FP-nZVI) at a certain time t was measured with a measuring cylinder and a stopwatch. The permeability coefficient (K) of the quartz sand (or FP-nZVI) was calculated using the following equation:

$$K=QL/Aht$$

where A is cross-sectional area of the column, h is the head difference between the bottom and the top of the column filled with quartz sand (or FP-nZVI).

Text S3. Dispersion coefficient experiment.

Column filled with quartz sand (or FP-nZVI) of height X. The sand column was fully saturated and cleaned with deionized water from bottom to top before the KCl solution was injected. The KCl solution with a concentration of 0.01 mol/L was continuously injected into the saturated sand column. The flow rates were 1, 3 and 5 mL/min, and samples were taken in the sampling hole at regular intervals to measure the conductivity value until the conductivity value reached stability. The C/C_0~t curves were plotted.

Find the time $t_{0.16}$ and $t_{0.84}$ corresponding to C/C_0 values equal to 0.16 and 0.84 respectively in the curve. Dispersion coefficient is calculated according to the following Equation:

$$D = \frac{1}{8}\left[\frac{X - U \cdot t_{0.16}}{\sqrt{t_{0.16}}} - \frac{X - U \cdot t_{0.84}}{\sqrt{t_{0.84}}}\right]$$

where D (cm^2/min) is the dispersion coefficient, X (cm) is the coordinate of the calculation point, U (cm/min) is the velocity of pore water in the column.

Text S4. Adsorption Experiment of Cr(VI) by quartz sand.

In order to analyze the physicochemical interaction between adsorbate and adsorbent, isothermal adsorption analysis was performed using quartz sand (30 g) or one piece of FP-nZVI prepared with 0.8 mol/L iron solution in 200 mL of different Cr(VI) concentrations (5, 10, 20, 30, 40, 50, 60 mg/L; 5, 10, 20, 30, 40 mg/L) at 25°C and pH=5, respectively. The medium equilibrium adsorption capacity is calculated by the following formula:

$$q_e = \frac{(C_0 - C_e)V}{W}$$

where q_e (mg/g) is the adsorption capacity of the medium in equilibrium, C_0 (mg/L)and C_e (mg/L) are Cr(VI) concentrations at initial and equilibrium, respectively, V (L) is the volume of solution, W (g) is the mass of the medium.

The adsorption isotherm data is described by the following models:

Langmuir model: $q_e = \frac{bK_LC_e}{1+K_LC_e}$

Freundlich model: $q_e = K_F C_e^{\frac{1}{n}}$

Temkin model: $q_e = blnK_T + blnC_e$

where K_L (L/mg) and K_F (L/g) are the adsorption coefficient of Langmuir and Freundlich models respectively, b (mg/g) is the maximum adsorption capacity of the medium to the solute, n is a constant, K_T (L/mg) is the equilibrium binding constant corresponding to the maximum binding energy, and constant b (KJ/mol) is related to the heat of adsorption.

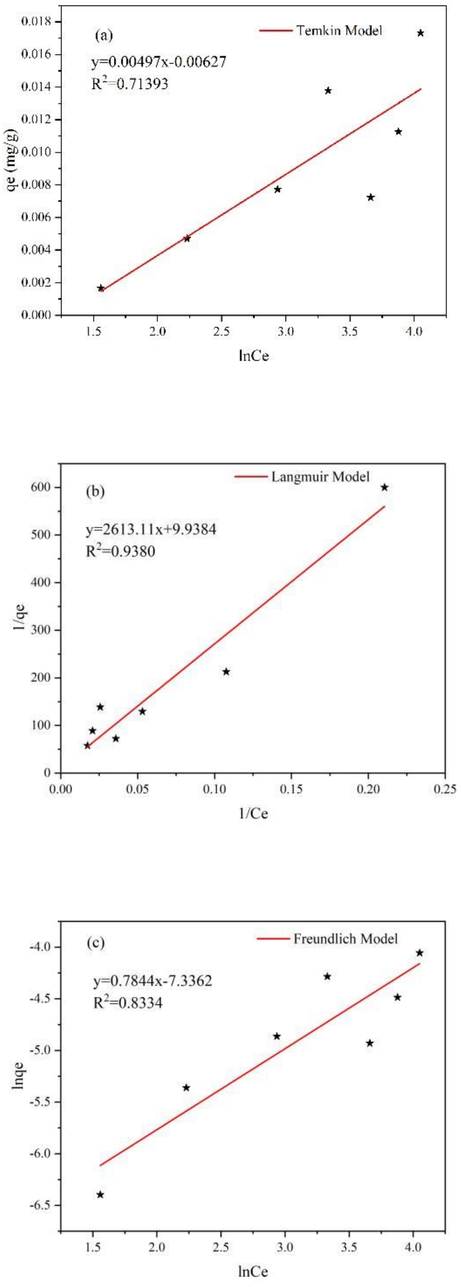

Figure S1. Adsorption Fitting Curve of Cr(VI) by quartz sand. (a) Temkin model; (b) Langmuir model; (c) Freundlich model.

Table S1. Porosity related parameters and results.

Medium	V_1 (cm^3)	V_2 (cm^3)	n
Quartz sand	113.04	57	0.5042
FP-nZVI	4.95	4.90	0.9908

Table S2. Permeability coefficient related parameters and results.

**Medium**	**Q（mL）**	**L（cm）**	**A（cm^2）**	**h（cm）**	**t（min）**	**K（cm/min）**
Quartz sand	8	7.5	7.065	0.6	3	4.7181
FP-nZVI	39	0.2	7.065	0.2	0.5	11.0403

Table S3. Dispersion coefficient related parameters and results.

Flow Rate	Medium	X（cm）	U（cm/min）	$t_{0.16}$ (min)	$t_{0.84}$ (min)	D（cm^2/min）
1	Quartz sand	4	0.2807	10.154	21.163	0.0978
	FP-nZVI	4	0.1429	9.694	60.926	0.1369
3	Quartz sand	4	0.8442	3.744	7.957	0.1744
	FP-nZVI	4	0.4286	3.101	19.790	0.2383
5	Quartz sand	14.5	1.4036	9.051	13.762	0.2369
	FP-nZVI	1	0.7143	3.143	20.500	0.2889

Table S4. Comparison of removal capacity for Cr(VI) by FP-nZVI with other adsorbents.

Adsorbent	pH	Temperature (°C)	q_{eq} (mg/g)	References
AC/nZVI	4	22	25	[S1]
AVT-nZVI	5	30	59.17	[S2]
ZVI-GAM	4	40	15.7	[S3]
NZVI/GNS	7	25	21.72	[S4]
NZVI/MMT	3	25	15	[S5]
PSA-nZVI	5.6	25	138.8	[S6]
CMC-nZVI@BC	7.5	25	80.71	[S7]
nZVI-BC	2	25	172.4	[S8]
FP-nZVI	5	25	19.76	This study

Reference

[S1] Mortazavian, S.; An, H.; Chun, D.; Moon, J., Activated carbon impregnated by zero-valent iron nanoparticles (AC/nZVI) optimized for simultaneous adsorption and reduction of aqueous hexavalent chromium: Material characterizations and kinetic studies. *Chemical Engineering Journal* **2018,** *353*, 781-795.

[S2] Zhao, R.; Zhou, Z.; Zhao, X.; Jing, G., Enhanced Cr(VI) removal from simulated electroplating rinse wastewater by amino-functionalized vermiculite-supported nanoscale zero-valent iron. *Chemosphere* **2019,** *218*, 458-467.

[S3] Liu, J.; Mwamulima, T.; Wang, Y.; Fang, Y.; Song, S.; Peng, C., Removal of Pb(II) and Cr(VI) from aqueous solutions using the fly ash-based adsorbent material-supported zero-valent iron. *Journal of Molecular Liquids* **2017,** *243*, 205-211.

[S4] Li, X.; Ai, L.; Jiang, J., Nanoscale zerovalent iron decorated on graphene nanosheets for Cr(VI) removal from aqueous solution: Surface corrosion retard induced the enhanced performance. *Chemical Engineering Journal* **2016,** *288*, 789-797.

[S5] Wu, L.; Liao, L.; Lv, G.; Qin, F., Stability and pH-independence of nano-zero-valent iron intercalated montmorillonite and its application on Cr(VI) removal. *J Contam Hydrol* **2015,** *179*, 1-9.

[S6] Jia, Z.; Shu, Y.; Huang, R.; Liu, J.; Liu, L., Enhanced reactivity of nZVI

embedded into supermacroporous cryogels for highly efficient Cr(VI) and total Cr removal from aqueous solution. *Chemosphere* **2018,** *199*, 232-242.

[S7] Zhou, H.; Ma, M.; Zhao, Y.; Baig, S. A.; Hu, S.; Ye, M.; Wang, J., Integrated green complexing agent and biochar modified nano zero-valent iron for hexavalent chromium removal: A characterisation and performance study. *Sci Total Environ* **2022,** *834*, 155080.

[S8] Huang, X.; Niu, X.; Zhang, D.; Li, X.; Li, H.; Wang, Z.; Lin, Z.; Fu, M., Fate and mechanistic insights into nanoscale zerovalent iron (nZVI) activation of sludge derived biochar reacted with Cr(VI). *J Environ Manage* **2022,** *319*, 115771.

Table S5. Isothermal model parameters of quartz sand to Cr(VI), standard error was in parentheses.

Medium	Temkin equation			Langmuir equation			Freundlich equation		
	K_T (L/mg)	b (KJ/mol)	R^2	K_L (L/mg)	b (mg/g)	R^2	K_F (L/g)	n	R^2
Quartz sand	0.28321 (0.5875)	0.00497 (0.0014)	0.71393	0.0038 (0.0009)	0.10062 (0.0812)	0.9380	0.00065 (0.0007)	1.2749 (0.5310)	0.8334
